# A Review of Additive Manufacturing of Biodegradable Fe and Zn Alloys for Medical Implants Using Laser Powder Bed Fusion (LPBF)

**DOI:** 10.3390/ma17246220

**Published:** 2024-12-19

**Authors:** Irene Limón, Javier Bedmar, Juan Pablo Fernández-Hernán, Marta Multigner, Belén Torres, Joaquín Rams, Sandra C. Cifuentes

**Affiliations:** 1 Department of Applied Mathematics, Materials Science and Engineering and Electronic Technology, Escuela Superior de Ciencias Experimentales y Tecnología (ESCET), Universidad Rey Juan Carlos, C/Tulipán s/n, 28933 Móstoles, Spain; irene.limon@urjc.es (I.L.); javier.bedmar@urjc.es (J.B.); juanpablo.fernandez@urjc.es (J.P.F.-H.); marta.multigner@urjc.es (M.M.); belen.torres@urjc.es (B.T.); joaquin.rams@urjc.es (J.R.); 2 Institute of Research on Technologies for Sustainability (ITPS), Universidad Rey Juan Carlos, C/Tulipán s/n, 28933 Móstoles, Spain

**Keywords:** biodegradable metals, iron alloys (Fe), zinc alloys (Zn), additive manufacturing (AM), laser powder bed fusion (LPBF), bioresorbable implants

## Abstract

This review explores the advancements in additive manufacturing (AM) of biodegradable iron (Fe) and zinc (Zn) alloys, focusing on their potential for medical implants, particularly in vascular and bone applications. Fe alloys are noted for their superior mechanical properties and biocompatibility but exhibit a slow corrosion rate, limiting their biodegradability. Strategies such as alloying with manganese (Mn) and optimizing microstructure via laser powder bed fusion (LPBF) have been employed to increase Fe’s corrosion rate and mechanical performance. Zn alloys, characterized by moderate biodegradation rates and biocompatible corrosion products, address the limitations of Fe, though their mechanical properties require improvement through alloying and microstructural refinement. LPBF has enabled the fabrication of dense and porous structures for both materials, with energy density optimization playing a critical role in achieving defect-free parts. Fe alloys exhibit higher strength and hardness, while Zn alloys offer better corrosion control and biocompatibility. In vitro and in vivo studies demonstrate promising outcomes for both materials, with Fe alloys excelling in load-bearing applications and Zn alloys in controlled degradation and vascular applications. Despite these advancements, challenges such as localized corrosion, cytotoxicity, and long-term performance require further investigation to fully harness the potential of AM-fabricated Fe and Zn biodegradable implants.

## 1. Introduction

### 1.1. Context and Background

Biodegradable metals have been defined as “metals expected to corrode gradually in vivo, with an appropriate host response elicited by released corrosion products, and dissolve completely upon fulfilling their role to assist with tissue healing, leaving no implant residues” [1]. Therefore, biodegradable metals must be composed of essential metallic elements that can be metabolized by the human body while demonstrating appropriate degradation rates and modes [1].

Research on biodegradable metals has primarily focused on magnesium alloys, with several commercial devices already available [2,3]. However, iron (Fe) and zinc (Zn) have emerged as particularly promising alternatives for resorbable implants due to their unique combination of mechanical properties, corrosion rates, and biological functions. Both Fe and Zn are essential elements involved in physiological processes, enhancing their suitability for medical applications such as cardiovascular stents, bone fracture fixation, and bone defect scaffolds. Table 1 presents the average amount present in the human body, blood serum levels, and daily allowances of Fe, Zn, and Mg, along with their mechanical properties and in vitro corrosion rates. Their ability to degrade in biological conditions, along with favorable mechanical properties, makes Fe and Zn promising candidates for biodegradable medical devices used in cardiovascular treatments, bone fractures, and bone defect repairs [4,5]. Fe’s high modulus and strength make it ideal for applications requiring load-bearing properties, while Zn’s intermediate corrosion rate and biocompatibility offer significant advantages in vascular and bone healing contexts.

Bioresorbable metals hold significant potential for enhancing the properties of current permanent implants and resolving their long-term complications. Applications of such metals include cardiovascular stents, internal fracture fixation implants, and scaffolds for bone defect regeneration.

**Figure 1:**
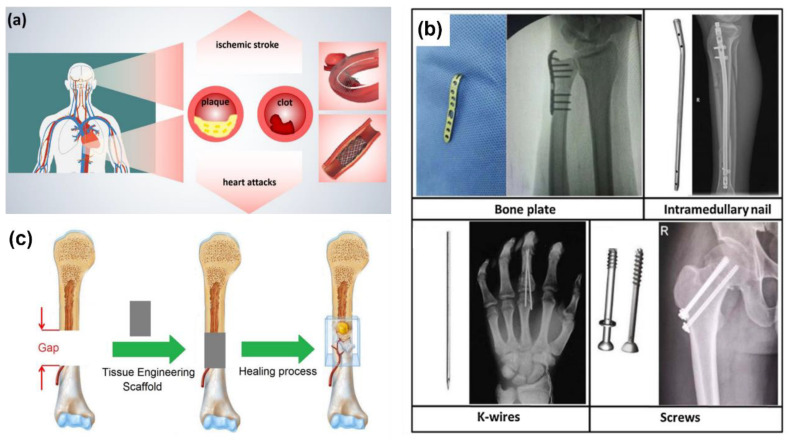
Applications of biodegradable metals in the medical devices industry. (**a**) Schematic diagram of cardiovascular diseases and stent [13]. (**b**) Common medical devices used for fracture internal fixation Adapted with permission from [14]. 2024 Elsevier. (**c**) Tissue engineering approach for reconstruction of large bone defects [15].

For cardiovascular stents (Figure 1a), which support the inner walls of blood vessels to treat obstructions, the need for a permanent implant is unnecessary as the support function is required for only about six months. Permanent stents can cause complications like late-stage thrombosis and chronic inflammatory reactions [6,7]. By contrast, resorbable stents can degrade after serving their purpose, thus avoiding long-term complications. These biodegradable stents must maintain radial force during the healing process, typically between 23 kPa and 40 kPa [8]. Furthermore, the degradation must be uniform and complete, without accumulating corrosion products or triggering cytotoxic effects [9].

For internal fracture fixation (Figure 1b), resorbable implants offer an alternative to permanent devices, which often require removal due to long-term issues like hypersensitivity, chemical carcinogenesis, and stress-shielding [4,10,11]. Resorbable osteosynthesis devices eliminate the need for removal surgeries while maintaining stability during bone healing. The degradation of such devices must be predictable and synchronized with the healing process to avoid adverse reactions [4].

Bone defect restoration (Figure 1c) also benefits from resorbable implants, as bone grafting with permanent materials may impede full regeneration [5]. In this context, bioresorbable metal-based scaffolds can provide the necessary mechanical strength and biocompatibility to support osteoconduction and bone healing without causing long-term issues associated with inert materials [12].

Fe-based materials have shown promise, especially for stents, given their high modulus, strength, and ductility, which make them suitable for wire fabrication. Nevertheless, Fe-based implants are still in the animal testing stage [16]. Zn, on the other hand, has attracted attention due to its corrosion rate, which lies between Fe and Mg, making it suitable for cardiovascular and bone applications. While Zn shows great promise, further in vivo studies are necessary for both Fe and Zn to progress to clinical trials [16].

A critical factor influencing the performance of biodegradable implants is the microstructure, which is dictated by both the alloy composition and manufacturing processes. Conventional manufacturing techniques for metallic implants, such as casting, machining, and powder metallurgy [13,17], often fall short in producing patient-specific designs or intricate geometries required for personalized medicine. Additive manufacturing (AM) addresses these limitations by enabling the fabrication of complex, patient-specific geometries with high precision and cost efficiency [18].

AM is a group of techniques that consists of a layer-by-layer fabrication of tridimensional parts from a previous design [19]. This kind of manufacturing allows the obtaining of complex pieces without an increment in the costs [18], which is not possible for most of the conventional processing techniques [20]. Moreover, AM can lead to near-net-shape parts without the need for molds and wasting of material [21], which also prevents the increments in costs. These advantages are important for the fabrication of personalized products, which is relevant for the biomedical sector [22]. AM, particularly laser powder bed fusion (LPBF), is already widely used in the medical industry to produce implants from bioinert materials like titanium, stainless steel, and CoCr alloys [17,23,24]. AM makes possible the fabrication of stents that fit the curvatures and sizes of blood vessels, screws, and plates that are conformed to the bone contour of the patient, or scaffolds with complex geometrical designs that mimic the structure of bone [13,17].

Applying AM to biodegradable metals introduces new challenges, particularly in controlling the microstructure and ensuring the mechanical properties and corrosion behavior meet clinical requirements. This review focuses on the advancements in AM, with a particular emphasis on LPBF for processing Fe and Zn biodegradable alloys. It explores how LPBF can optimize the microstructure, mechanical performance, and corrosion behavior of these materials to meet clinical requirements. The review also examines the biocompatibility of Fe and Zn alloys in both in vitro and in vivo settings, aiming to advance their development as next-generation bioresorbable implants for medical applications.

### 1.2. PRISMA

**Figure 2:**
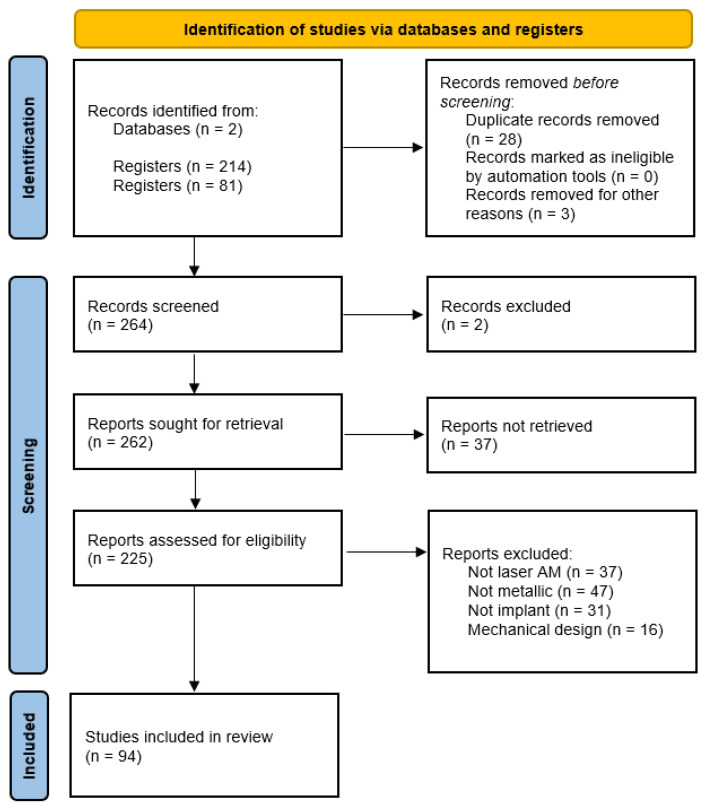
PRISMA flow diagram used for reporting systematic reviews [25].

The preferred reporting items for systematic reviews and meta-analyses (PRISMA) method was adopted for this study. The methodology consists of four systematic steps: (i) identification; (ii) screening; (iii) eligibility; and (iv) qualitative synthesis. These steps are summarized in Figure 2. Based on PRISMA guidelines, the identification process in this study focused on publications (articles, proceedings, and reviews) from the Web of Science (WoS) and Scopus databases. The final search was performed in September 2024, with the time frame set to include publications up to March 2024. Since the technologies under investigation are relatively recent, no initial time constraint was set.

In both queries, the search string included the following search terms and Boolean operators: (Fe OR Zn OR Iron OR Zinc) AND (Additive Manufacturing OR SLM OR LPBF) AND (Biomaterial). The results obtained included 214 from the WoS and 81 from Scopus, with 28 duplicates. An initial screening was then conducted, removing non-relevant articles. Articles that focused on theoretical mechanical evaluations, oxides, chemical compounds of the metals, or were not related to biomaterials were excluded from the dataset.

This was followed by a second screening process, which involved a more detailed analysis of the abstracts (225 remaining articles). The articles were then categorized based on their primary subject. The final dataset contained 94 documents, which were then used to synthesize this review.

### 1.3. Additive Manufacturing—Laser Powder Bed Fusion

Powder bed fusion techniques involve the deposition of a layer of powder, which is spread across a powder bed and subsequently melted or sintered using a laser or an electron beam [26]. This family of processes is the most complex among additive manufacturing techniques due to the large number of manufacturing parameters, the advanced technology of the printer, and the overall complexity of the manufacturing process.

With this technology, it is possible to print parts with complex geometries, but at much slower speeds compared to other additive manufacturing processes, such as direct energy deposition (DED). The powder bed fusion family includes techniques such as laser powder bed fusion (LPBF), selective laser sintering (SLS) [27,28], or electron beam melting (EBM) [29], which differ from each other based on the energy source used to process the metal powder or the effect they have on it.

Regarding the use of LPBF in medical devices, this technique has been applied to several inert metals for the fabrication of biomaterials (e.g., Ti6Al4V, 316L stainless steel, cobalt-chromium, etc.). Compared to other additive manufacturing (AM) techniques, such as direct energy deposition (DED), LPBF is capable of fabricating more complex geometries, which is important for the personalization required in such applications. Additionally, porous biomaterials, which are crucial in the field of biomaterials, can be easily fabricated using LPBF.

In the LPBF technique, the energy source used is a laser that melts the metal powder distributed over a bed of the same material, performing layer by layer, melting and remelting of the metal. This technique allows the manufacture of parts with shapes close to those of the final parts (near-net-shape), with high densities, complex geometries, and excellent mechanical properties. However, it involves controlling a large number of parameters, such as scan speed, build direction, angle of rotation of the laser scan between layer (also known as the infill angle), laser power, and the possibility to overlap each layer’s edge, among others.

The laser is a crucial tool in this type of manufacturing and is classified into three main types: diode lasers, fiber lasers, and CO_2_ lasers. This review examines scientific studies that utilized one of these three laser types. Diode lasers offer several advantages, including low cost, minimal maintenance, low energy consumption, homogeneous beam intensity distribution, and high absorptivity for metals. However, their beam has low optical quality, which limits the laser’s focusing ability and prevents the production of highly detailed structures [30]. Fiber lasers, on the other hand, are the most widely used in laser powder bed fusion techniques. Their gain medium is a rare earth-doped optical fiber that emits in the near-infrared range. These lasers are known for their high beam quality, which stems from their optical components and fiber-based gain medium. Additionally, they are robust against environmental disturbances and feature compact systems. Their high efficiency allows for the production of parts with fewer defects compared to other types of lasers [31]. Lastly, CO_2_ lasers have been the most commonly used industrial lasers for years. These lasers operate by electrically pumping a gas with direct or alternating current to induce the population inversion needed for lasing. CO_2_ lasers provide high efficiency (20%) and output power ranging from 0.1 to 20 kW. They are relatively simple, reliable, and cost-effective, making them suitable for applications such as cutting, welding, marking, and surface modification. However, their primary drawback is their emission wavelength of 10.6 μm, which is more difficult to control. This results in beams with greater divergence and a larger focal spot size [32]. Additionally, CO_2_ lasers have low absorptivity by many metals [31]. Moreover, the light in these lasers travels through a path made of mirrors, which results in power losses and reduces the energy delivered to the powder.

The atmosphere is another important factor to control in metal 3D printers. Some metals used in the biomedical sector have very low vapor pressures, which can lead to sublimation. Magnesium and zinc are the most common biometals affected by this phenomenon. To prevent this issue, LPBF equipment must have precise atmospheric control to ensure proper pressure inside the printer. Printers such as EOS or Acconity are equipped with systems that regulate pressure, making them suitable for fabricating metals like Zn.

Other parameters that should be considered to optimize the manufacturing process of parts are the intrinsic properties of the material. Table 2 summarizes the properties of pure resorbable metals (Zn, Fe, and Mg) related to LPBF processing. Absorptivity, melting point, and specific heat should be taken into account to conduct a thorough parameter study and to link processing variables with material properties. Depending on the values of these parameters, the material’s energy absorption can be increased or decreased. For example, materials with low melting points or high absorptivity are more susceptible to defects caused by excessive energy input [33]. Therefore, it is important to control the energy input received by the metal by calculating it from the manufacturing parameters. High energy input can lead to defects such as vaporization of the molten metal or the formation of cracks during solidification. Conversely, low energy input can result in defects related to lack of fusion [34].

**Table 2:** Properties of Zn, Fe, and Mg related to LPBF processing [33].

Properties	Unit	Value		
		Zn	Fe	Mg
Density (20 °C)	g/cm^3^	7.14	7.874	1.74
Melting point	°C	419.5	1538	650
Boiling point	°C	907	2862	1091
Heat conductivity (20 °C)	W/m·K	113	80	158
Heat conductivity (melting point)	W/m·K	61	40	78
Specific heat (20 °C)	J/kg K	382	444	1360
Surface tension (melting point)	mN/m	782	1835	559
Viscosity (melting point)	mPa·s	3.85	6.93	1.25
Laser absorptivity (powder, 20 °C)	%	70	75	/

LPBF systems have a large number of manufacturing parameters that can be controlled. The principal ones are described in the following paragraphs. Most of these parameters are related to the laser, which is the key element in this type of fabrication.

Power: Laser power is an intrinsic parameter that depends on the type of laser used during fabrication. Although laser power can be adjusted within a certain range, some materials have a narrow working window due to their low vapor pressure, meaning that high power can cause sublimation of the material. On the other hand, low laser power can lead to defects caused by insufficient energy during printing, such as pores, unmelted particles, and lack of fusion [34]. Certain types of lasers, such as CO_2_ lasers, have low absorption by metals, whereas fiber lasers offer better absorption.

Scan speed: Scan speed is a more flexible parameter than power, making it one of the most studied for optimizing the fabrication of different metals. In general, scan speed refers to the velocity at which the laser sweeps across the powder bed. Scan speed significantly affects the energy input to the metal. A high scan speed results in low-energy contribution to the powder bed, leading to defects like voids. A lower scan speed can reduce these defects, but it must be carefully controlled to avoid problems such as metal sublimation or hot cracking [34]. Scan speed is usually expressed in millimeters per second (mm/s).

Layer height: An important step in LPBF fabrication is moving the powder bed each time a new layer is spread. Since the laser focus is fixed, the powder bed needs to move down when a new layer is added to keep the focus on the previous layer. A key decision is the layer height, which refers to the distance the powder bed moves to maintain the laser focus on the powder bed. Depending on the material and its relation to other parameters, the layer height typically ranges from 20 to 60 µm.

**Figure 3:**
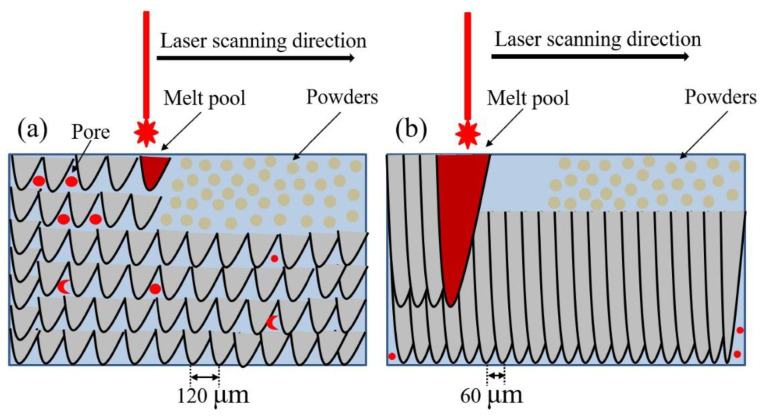
Scheme from Li et al. showing the LPBF process at hatch distances of 120 μm (**a**) and 60 μm (**b**) Reprinted with permission from [35]. 2024 Elsevier.

Hatch distance: This parameter is the distance between the focal point of the laser track and the closest focal point of the next track within the same layer. It is linked to the overlap between adjacent scanning tracks and plays a significant role in the densification of the metal [35]. A larger hatch distance can lead to defects, such as lack of fusion, with unmelted particles appearing in the final part. Hatch distance is usually around 100 µm, and reducing this parameter improves densification. A diagram of the effects of different hatch distances can be seen in Figure 3.

Beam radius: The beam radius refers to the area where the laser is fully focused on the powder bed. This parameter must be carefully studied to establish a good relationship between it and other parameters, such as hatch distance, to ensure an optimal combination of both.

The parameters of the LPBF technique influence the relative density, microstructure, and mechanical properties of the materials. These parameters are constrained by the performance of the equipment, including technical factors such as laser power, hatch distance, and scanning speed can vary.

The energy equation governing the process is as follows:

(1) $$E_{v}=\frac{P}{h\times v\times z}$$

where *E_v_* is the volumetric energy density, *P* is the power (W), *h* is the hatch distance, *v* is the scanning speed, and *z* is the layer thickness. The energy input into the material powder significantly influences the microstructure and densification.

Depending on the energy input, the manufactured parts can have different types of defects. When the energy input is lower than necessary, one of the resulting problems is the presence of voids, which can be classified into two groups [36]. The first group is porosity, caused by gas entrapment during the solidification of the molten metal. The origin of this gas can vary, ranging from the atmosphere used during printing to gas previously trapped in the starting metal, particularly in the case of powders. The second group is lack of fusion, which occurs due to insufficient applied energy on the starting metal. Another defect caused by insufficient energy is delamination, which involves poor welding between layers. This can result in the loss of integrity, with the final part separating into several sublayers. Poor welding can also lead to weak joints between the part and the printer base, potentially causing the part to detach. On the other hand, when energy inputs are too high, the primary issue is material vaporization, particularly due to the low vapor pressure of certain materials.

## 2. Additive Manufacturing of Iron-Based Alloys

Iron (Fe) is an essential trace element for human life and has attracted considerable research interest in the development of biodegradable implants over the past two decades. Its main advantages are its higher mechanical properties and ease of formability compared to other metals used in biodegradable medical applications, such as magnesium (Mg) and zinc (Zn). Additionally, Fe demonstrates reasonable biocompatibility, but it has a very low corrosion rate, which is often insufficient for vascular and bone applications. Various strategies have been addressed to increase the degradation rate of pure Fe, including alloying with other elements [37], addition of secondary phases [38], or microstructure refinement [39], which in turn are carried out with several processing techniques [40].

Among Fe alloys, FeMn, containing varying proportions of manganese (Mn), has likely generated the most interest [41,42], and some alloys even include additional elements such as carbon (C) and palladium (Pd) [43]. The addition of Mn increases the corrosion rate and enhances the mechanical properties of Fe, particularly strength and elongation. Mn also helps stabilize non-ferromagnetic phases, which is relevant for the use in magnetic resonance imaging (MRI) systems. However, Mn may pose toxicity risks [44].

The in vivo biocompatibility of Fe and Fe alloys has been demonstrated in various animal models [42,45]. However, in vitro biocompatibility results remain inconsistent. Eluted tests indicate acceptable biocompatibility, whereas cytotoxicity is observed in direct-contact assays [46]. Corrosion products of Fe and its alloys are poorly soluble in saline solutions, which can negatively impact cell culture tests due to the static conditions involved.

Regarding applications, most research is focused on the cardiovascular field, particularly on stents [3,46]. Fe’s high elastic modulus reduces elastic recoil, and its combination of high strength and elongation enables the design of stents with thinner struts than polymeric or Mg counterparts, achieving comparable mechanical performance. Thinner struts help minimize arterial wall injury during deployment, improve hemodynamics, and reduce the release of corrosion products. Notably, ultrathin strut (≈70 mm) Fe-based stents have shown promising in vivo results [47,48]. For bone applications, research on Fe alloys is primarily focused on developing scaffolds for bone tissue regeneration using traditional powder metallurgy techniques [49] and, more recently, advanced techniques like additive manufacturing [50]. However, further in vitro and in vivo studies are required to fully assess the feasibility of Fe for this application.

### 2.1. Linking Processing Parameters and Energy Density to Densification

**Figure 4:**
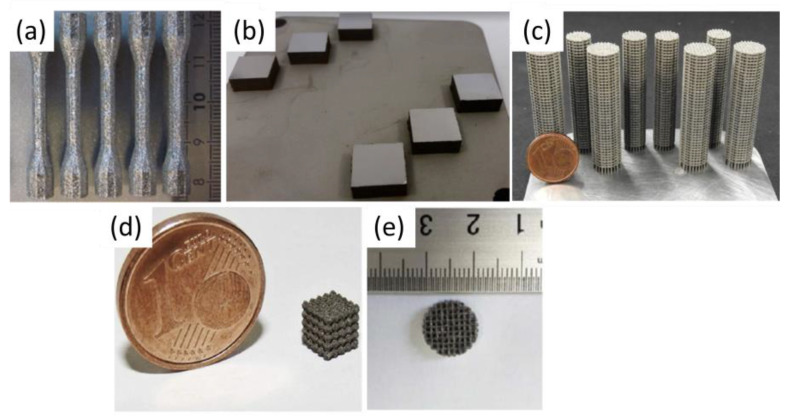
Additive manufactured parts: (**a**) Adapted with permission from [51], 2024, Elsevier (**b**) [56], (**c**) Adapted with permission from [53] 2024, Elsevier, (**d**) Adapted with permission from [54] 2024, Elsevier and (**e**) [55].

**Figure 5:**
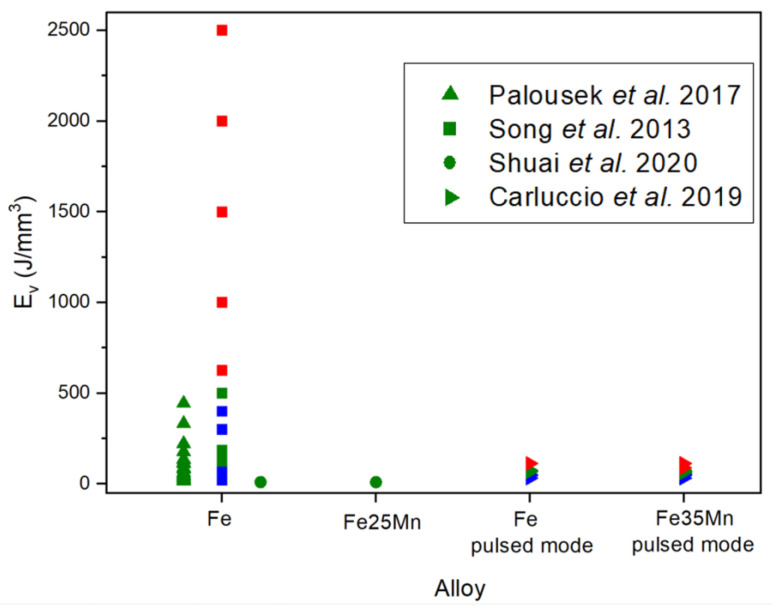
Parts quality according to *E_v_* and material. Green indicates optimum conditions, red excess of energy, and blue lack of energy [51,52,53,54,55,56].

In most studies on additive manufacturing of Fe, fiber lasers are used, with energy densities ranging from 10 to 2500 J/mm^3^ [51,52] for pure iron and lower energy densities, between 10 and 113 J/mm^3^, for its alloys [53,54,55]. Figure 4 shows additive manufactured Fe-based parts, including dense parts (a and b) and scaffolds (c–e). Figure 5 illustrates part quality in relation to process energy density, the types of alloys used, and laser working modes, including continuous wave (CW) and pulsed wave (PW) mode. Blue indicates typical defects from low-energy density, green represents high-quality parts, and red indicates defects caused by excessive energy. The consideration of these colors was taken in accordance with the conclusions of the authors. The energy density (J/mm^3^) for CW mode was calculated using Equation (1), while PW mode energy density was calculated using Equation (2).

(2) $$E_{v}=\frac{P\times t_{on}}{d_{p}\times h\times z}$$

where *P* is the laser power (W), *v* is the scan speed (mm/s), *h* is the hatch distance (mm), *z* is the layer thickness (mm), *t_on_* is the pulse duration (s), and *d_p_* is the point distance (mm).

**Figure 6:**
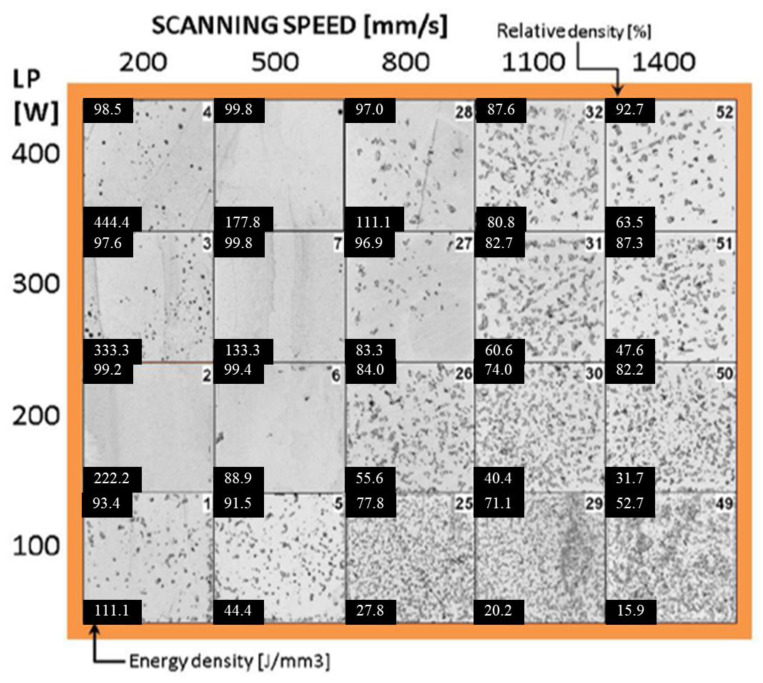
Preliminary study of processing parameters for hatch distance of 90 µm. The number at the top left is the relative density (%), the top right is the number of the sample and the number at the bottom left is the energy density (J/mm^3^) [56].

Palousek et al. [56] studied the process parameters for producing dense, pure iron parts. The parameters included laser power (100–400 W), laser scanning speed (0.2–1.4 m/s), and hatch distance (90–150 µm), while keeping the layer thickness constant at 50 µm. The energy density range used in this work is represented by triangles (**▲**) in Figure 5. Slower scanning speeds (0.2 and 0.5 m/s) resulted in parts with high relative densities (80–99%), whereas higher scanning speeds (0.8 and 1.4 m/s) produced parts with lower relative densities (approximately 45–97%). These results are shown in Figure 6, which displays the porosity analysis as a function of scan speed and laser power at a constant hatch distance (90 µm). However, their goal was to identify a process window that balanced quality with cost. Since lower scan speeds increase manufacturing time and thus the cost of each part, these speeds were excluded from further analysis. The optimal process window was established between 0.5 and 0.8 m/s and 400 W of laser power, with 400 W and 0.75 m/s chosen to maximize productivity.

**Figure 7:**
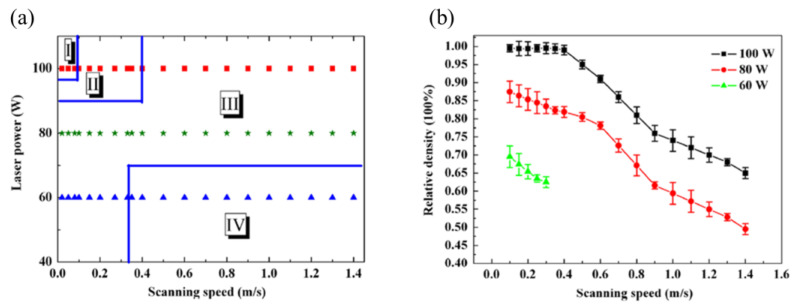
(**a**) Processing windows for pure iron (Zone I—Deformation zone, Zone II—Formation zone, Zone III—Zone of poor formation, and Zone IV—Zone of non-forming) versus laser power and scanning speed; Red square: parts manufactured with 100 W of laser power, green asterisks 80 W and blue triangles at 60 W. (**b**) Density curves of iron parts as a function of the laser power and scanning speed Adapted with permission from [51]. 2024, Elsevier.

Song et al. (represented in Figure 5 as squares) [51] investigated different parameters for pure iron, with energy densities ranging from 20 to 2500 J/mm^3^. They varied the laser power (60, 80, and 100 W) and scan speed (0.02–1.4 m/s) while keeping hatch distance (40 µm) and layer height (50 µm) constant. Figure 7a shows the parameters and resulting part quality in Song et al.’s study, while Figure 7b represents the part’s density according to laser power and scan speed. Their findings showed that highly dense parts (nearly 100% of density) were achieved only with a laser power of 100 W and a scan speed between 0.1 and 0.4 m/s. Lower laser powers (60 and 80 W) or higher scan speeds (0.4–1.4 m/s) led to defects such as delamination, brittle fractures, and high porosity [51]. Conversely, using high laser power (100 W) with low scan speeds (0.02–0.1 m/s) introduced excessive energy, resulting in high thermal stress and part deformation.

Shuai et al. [55] fabricated high-density Fe and Fe25Mn scaffolds in CW mode, using the same parameters for both materials. Their parameters included a laser power of 120 W, a scan speed of 3 m/s, a layer thickness of 50 µm, and a hatch distance of 40 µm. The energy density used in this study (20 J/mm^3^) was approximately ten times lower than that used by Song et al. as represented by circles in Figure 5. With these parameters, Shuai et al. successfully fabricated parts with no significant pores or cracks, indicating a high level of densification.

Carluccio et al. [52,53,54] worked in PW mode, fabricating dense pure iron parts and scaffolds of pure iron and Fe35Mn alloy. The energy density of both types of pieces is represented in Figure 5 as a right-facing triangle (►). For dense pure iron parts, a density of 99.2% was achieved with a laser power of 200 W, a pulse duration of 100 µs, a point distance of 60 µm, a hatch distance of 100 µm, and a layer thickness of 50 µm. The energy density for the dense parts was 67 J/mm^3^. For the scaffolds, the study varied the laser power (100–150 W), pulse duration (40–60 µs), and hatch and point distances (40–60 μm), while keeping the layer thickness constant at 50 µm. The highest quality of scaffolds was achieved with a laser power of 150 W, a pulse duration of 60 µs, and a hatch and point distance of 50 µm for pure Fe. The optimum parameters for Fe35Mn were a laser power of 125 W, a pulse duration of 50 µs, and a hatch and point distance of 45 µm. They attributed the difference in the parameters (energy density of 72 and 62 J/mm^3^, respectively) to the higher melting point (T_m_) of pure Fe, which made it more difficult to process into scaffolds compared to Fe35Mn.

From a general perspective, the optimal energy density range for iron and its FeMn alloys is not clearly defined, but the fabrication of scaffolds requires lower energy densities than the fabrication of dense parts. The choice of parameters appears to be crucial: a minimum laser power of 100 W seems effective for both dense parts and scaffolds in both the CW and PW modes. Additionally, scaffolds can be manufactured with higher scan speeds (0.8–1 m/s) compared to dense parts (0.1–0.6 m/s). In the case of dense parts, using high-energy densities (600–2500 J/mm^3^) results in thermal stress, leading to sample deformation, while for lattice parts, high input energy (89–113 J/mm^3^) reduces the designed porosity by forming larger melt pools and thicker struts [51,54]. On the other hand, using low-energy densities (20–100 J/mm^3^) in dense parts leads to poorly formed zones with defects like delamination, brittle fracture, high porosity, or even failure to form parts. For scaffolds (32–60 J/mm^3^), low-energy densities result in pores typical of partial powder fusion [51].

### 2.2. Influence of Microstructure on Hardness

In the LPBF process, the resulting microstructure typically consists of columnar grains that grow preferentially in the build direction, primarily due to the thermal gradient [57]. However, in the case of pure iron, some researchers have observed a microstructure predominantly composed of equiaxed grains, with only a few elongated columnar grains [52,54]. This can be attributed to the fact that the final α-ferrite grains seen in the microstructure are formed from two preceding solid phases. During cooling, the liquid phase first solidifies into delta iron, which subsequently transforms into austenite, and finally, the ferrite grains are formed (L → δ → γ → α) [52,54]. Song et al. [51] further noted that grain size decreases progressively with increasing scanning speed at a constant laser power.

**Figure 8:**
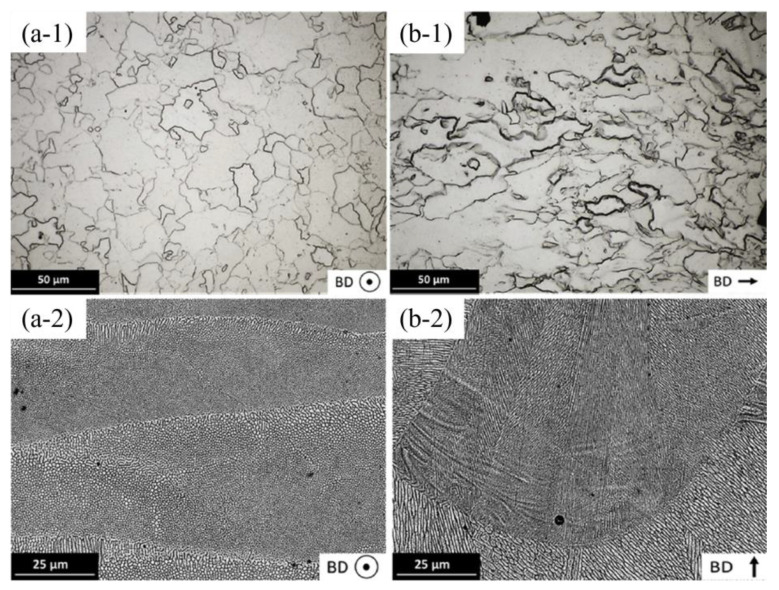
Pure Fe cross-sectional optical micrographs (**a-1**,**b-1**) and Fe35Mn SEM images (**a-2**,**b-2**) showing the microstructure with BD out-of-plane and in-plane. Adapted with permission from [54]. 2024, Elsevier.

In the case of FeMn alloys, different results have been observed depending on the Mn content. Carluccio et al. reported that the microstructure of Fe35Mn differs from that of pure Fe. They found that the addition of 35 wt% Mn stabilizes the γ-austenite phase and preserves the solidified microstructure produced during LPBF (L → γ). This results in a microstructure of large columnar γ-grains, with each γ-grain consisting of a network of individual cells. Figure 8 illustrates that Fe35Mn grains are finer than pure Fe grains [53,54].

Shuai et al. manufactured a bone scaffold using LPBF with an Fe25Mn alloy. Their results showed a microstructure characterized by uniform equiaxed grains containing α-Fe, a martensitic phase, and an austenitic phase, along with smaller grain sizes compared to pure iron parts printed under identical conditions [55]. Figure 8 further highlights the microstructural differences between pure iron and Fe35Mn.

In terms of hardness, Carluccio et al. [54] noted that, in accordance with the Hall–Petch relationship [58], the hardness of a polycrystalline metal increases as grain size decreases. Consequently, the α-ferrite in pure Fe is softer than the austenite in Fe35Mn, resulting in a lower overall hardness of 150 ± 6.5 Hv for pure Fe compared to 163 ± 4 Hv for austenitic Fe35Mn. Table 3 compares the microhardness values of materials from the Carluccio et al. study with the Fe-based biocompatible alloy AISI 316 L and other biodegradable metals such as Zn and Mg. The hardness values for the LPBF-processed materials are also compared with those produced via other manufacturing methods with human bone. The authors concluded that pure Fe and Fe35Mn alloy fabricated by additive manufacturing exhibited characteristics comparable to rolled mild steel, as their hardness surpassed that of LPBF-produced pure Zn and pure Mg.

**Table 3:** Comparison of Vickers microhardness (HV) values of biodegradable metals and AISI 316L produced by different manufacturing methods compared to human bone. n/a, not available [54].

Human Bone	40–79		
Material	SLM	Cast	Wrought
Pure Fe	150 ± 6.5	130 (Mild steel)	150 (Mild steel)
Fe35Mn	163 ± 4.0	n/a	n/a
AISI 316L	245 ± 6.0	175	220
Pure Zn	45 ± 5.4	n/a	34 ± 2
Pure Mg	78 ± 8.2	30 ± 2	40 ± 2

### 2.3. Mechanical Properties of Dense Structures and Scaffolds

The mechanical properties of pieces fabricated by LPBF, including both dense structures and scaffolds, have been tested by various authors, with results summarized in Table 4 and Table 5. Table 4 highlights the mechanical properties of dense Fe and FeMn alloys, comparing them to human cortical bone and cast Fe and FeMn alloys. Table 5 presents the mechanical properties of Fe and FeMn alloy scaffolds.

Song et al. [51] observed that, for dense pure iron, increasing the laser scan speed (i.e., decreasing the energy density) led to an increase in yield strength and tensile strength. They reported a yield strength of 245.87 MPa for an energy density of 185 J/mm^3^, and 305.37 MPa for an energy density of 125 J/mm^3^. This effect was attributed to grain size, as smaller grains were obtained with higher scan speeds. Carluccio et al. [52], using a lower energy density of 67 J/mm^3^ than Song et al., achieved an even higher yield strength of 421 MPa. These findings indicate that the LPBF process produces higher yield strength compared to casting. This suggests that the manufacturing process plays a critical role in determining yield stress. Song et al. [51] proposed that the overall strength of α-Fe can be contributed to the following formula:

(3) $$\sigma ={∆\sigma }_{gr}+{∆\sigma }_{wh}+{∆\sigma }_{ps}$$

where Δ*σ_gr_* is the yield strength due to grain refinement, Δ*σ_wh_* is the yield strength due to work hardening, and Δ*σ_ps_* is the yield strength due to secondary phase precipitation, being the grain refinement the most significant factor, followed by work hardening. The Hall–Petch relation states that the yield strength contribution from grain refinement is inversely proportional to the grain size [59]. A finer microstructure restricts dislocation motion due to the higher number of grain boundaries. The contribution to overall strength from work hardening is proportional to the dislocation density, which is further enhanced by the high cooling rates characteristic of the LPBF process. Both Song et al. and Carluccio et al. concluded that grain refinement is the most significant strengthening mechanism, followed by work hardening. Carluccio et al. quantified the contributions to overall strength, estimating that grain refinement accounts for approximately 87.5%, work hardening 12.25%, and secondary phase precipitation 0.25%.

**Table 4:** Mechanical properties of dense pure iron and its alloys.

Material	Energy Density (J/mm^3^)	E (GPa)	σ0.2 (MPa)	UTS (MPa)	Compressive Strength at 20% Strain (MPa)	Ref.
Human cortical bone	-	1–35	1–20		103–140	[53]
Cast Fe	-	202.5 ± 6.70	157.1 ± 7.7		497.8 ± 7.5	[52]
Cast Fe35Mn	-		240		440	[53]
SLM pure Fe	185	205.67 ± 16	245.87 ± 17	354.27 ± 18	-	[51]
SLM pure Fe	152	208.77 ± 16	256.57 ± 17	356.67 ± 22	-	
SLM pure Fe	143	210.57 ± 18	285.47 ± 20	402.77 ± 24	-	
SLM pure Fe	125	215.87 ± 20	305.37 ± 22	411.57 ± 25	-	
SLM pure Fe	67	199.70 ± 6.70	421.1 ± 16	-	760.2 ± 6.5	[52]

**Figure 9:**
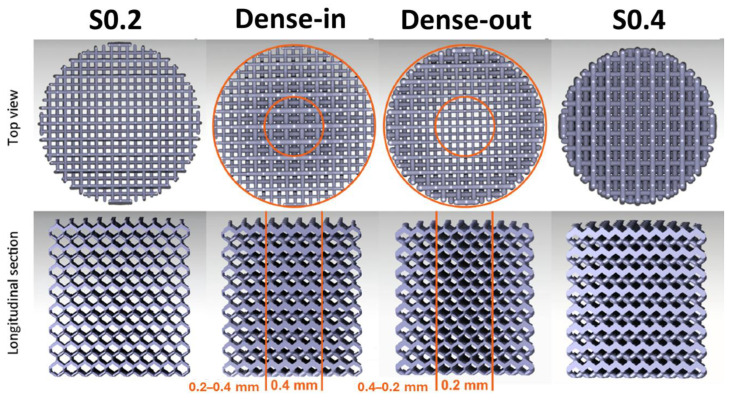
Top view and longitudinal cross-section of the CAD models of functionally graded Fe scaffold. Adapted with permission from [60]. 2024, Elsevier.

Regarding scaffolds manufactured by LPBF, Li et al. [60] studied various types of structures: a uniform structure with a 0.2 mm strut thickness (S0.2), a functionally graded porous structure with a strut thickness increasing from 0.2 mm at the periphery to 0.4 mm at the center (Dense-in), a functionally graded porous structure with a strut thickness decreasing from 0.4 mm at the periphery to 0.2 mm at the center (Dense-out), and a uniform porous structure with a 0.4 mm strut thickness (S0.4). Figure 9 shows the top and longitudinal views of these Fe scaffolds. The highest yield strength (53 MPa) was observed for the scaffold with a 0.4 mm strut thickness, while the lowest yield strength (10.7 MPa) corresponded to the 0.2 mm strut thickness. The functionally graded scaffolds, Dense-in and Dense-out, exhibited similar yield strength values of 33 MPa and 30.5 MPa, respectively.

**Table 5:** Mechanical properties of scaffolds pure iron and its alloys.

Material	Energy Density (J/mm^3^)	Struct Size (µm)/Pore Size (µm)	Geometry	E (GPa)	σ0.2 (MPa)	UTS (MPa)		Ref.
LPBF pure Fe		400/600	Diamond	2.82 ± 0.17	53.1 ± 0.9		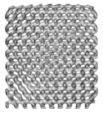	[60]
LPBF pure Fe		200/800	Diamond	0.89 ± 0.09	10.70 ± 0.40		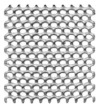	[60]
LPBF pure Fe		200–400/800–600	Diamond	1.77 ± 0.05	32.9 ± 1.6		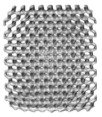	[60]
LPBF pure Fe		400–200/600–800	Diamond	1.75 ± 0.03	30.5 ± 0.3		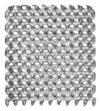	[60]
LPBF pure Fe	10	600–800/-	-		70.3 ± 4.2	135 ± 5.2	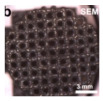	[55]
LPBF Fe25Mn	10	600–800/-	-		137 ± 8.4	221.7 ± 10.9	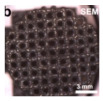	[55]
LPBF Fe35Mn	62	600/400	Primitive Surface p-unit	33.5 ± 1.70	89.2 ± 1.9	-	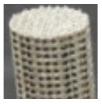	[53]

Shuai et al. [55] manufactured pure iron and Fe25Mn scaffolds using the same energy density and conducted compression tests to evaluate their mechanical properties. They observed that the FeMn alloy exhibited a higher yield strength (137 MPa) and ultimate strength (221.7 MPa) compared to the pure iron scaffolds (70.3 MPa and 135 MPa, respectively). This led them to conclude that alloying with Mn significantly enhances the compressive properties of the material.

Carluccio et al. [53] also studied the mechanical properties of Fe35Mn scaffolds through compression tests. They recorded a yield strength of 89.2 MPa and concluded that LPBF-manufactured Fe35Mn scaffolds exhibit suitable mechanical properties, particularly for highly loaded bone scaffold applications such as the mandible, which has been shown to have higher compressive strength than bones in other areas [61].

These investigations demonstrate that scaffolds generally achieve lower yield strength than dense samples. For dense parts, both yield strength and ultimate strength increase as energy density decreases. In the case of scaffolds, Fe25Mn and Fe35Mn alloys both exhibit higher yield strength than pure iron scaffolds, though Fe25Mn achieves a higher yield strength than the Fe35Mn alloy. This suggests that there may not be a direct correlation between yield strength and Mn content.

Higher yield strength and ductility are desirable for biomedical implants, as the mechanical properties degrade along with the implant [1]. During degradation, it is critical that the implant continues to provide support to the healing tissue. This is particularly important for porous medical implants like bone scaffolds, where high porosity can significantly reduce strength, further exacerbated by higher degradation rates [52].

### 2.4. Corrosion Behavior

**Figure 10:**
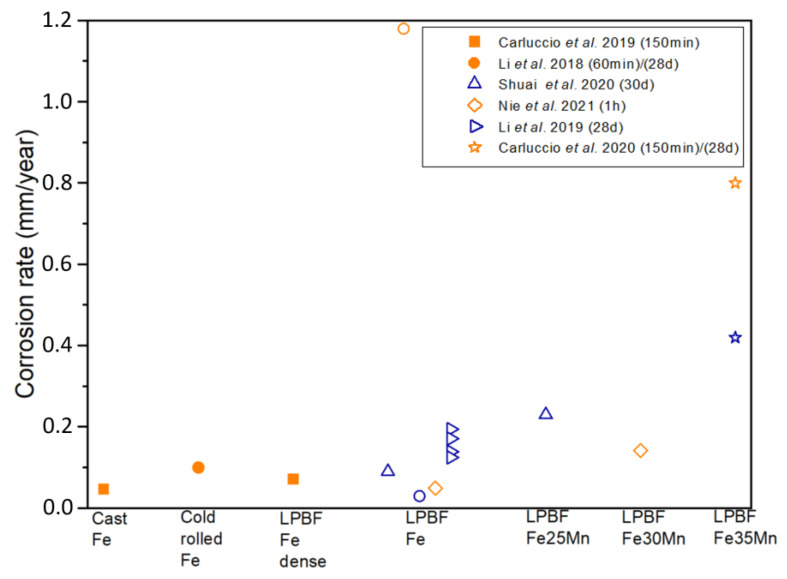
Corrosion rate of Fe and its alloys. In orange electrochemical tests and in blue immersion tests. Filled symbols correspond to dense parts and empty symbols correspond to scaffolds [52,53,55,60,63,64].

The corrosion rate of pure Fe is significantly lower compared to other biodegradable metals such as Mg and Zn. In general, when pure Fe is used in the manufacture of biodegradable devices, various treatments and strategies are employed to increase its degradation rate, making the material more suitable for the intended medical treatment duration. Thus, many studies have been carried out with the aim of increasing its degradation rate [62]. In the scientific literature, the in vitro corrosion behavior of Fe is typically assessed in terms of biodegradability, mass loss, and electrochemical properties, using an electrolyte that simulates biological conditions as the corroding medium. Investigations that have performed corrosion studies of LPBF-manufactured Fe parts and their alloys are summarized in Table 6 and are compared with iron pieces fabricated by other manufacturing methods. Conditions of the corrosion tests and the corrosion rate obtained from these studies are also shown in that table. Figure 10 shows the corrosion rates, in millimeters per year, of the samples evaluated in these studies, obtained through different tests. The corrosion rates from electrochemical tests are depicted in orange, while the corrosion rates from mass loss of the samples immersed in the simulated body fluid (SBF) electrolyte, simulating biological conditions, are shown in blue. Furthermore, the filled symbols correspond to dense parts, and empty symbols correspond to scaffolds. The authors of the studies and the duration of the tests appear in the legend. When comparing studies, it is important to ensure that all parameters used in the investigations are as similar as possible to obtain meaningful comparisons. Factors such as the type of electrolyte, temperature, pH, and scan rate in electrochemical tests play a crucial role in influencing corrosion results. These variables can significantly alter the corrosion behavior of materials and must be carefully controlled or accounted for. To address this variability, key parameters from the studies are summarized in Table 6 for clarity and consistency.

**Figure 11:**
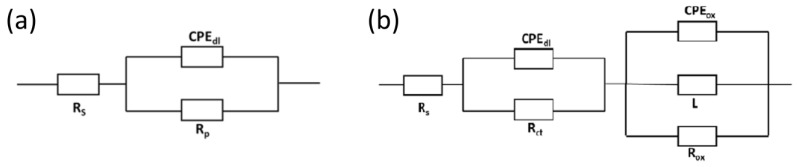
Circuit for (**a**) pure iron. Adapted with permission from [52] (2024, Elsevier) and (**b**) Fe35Mn scaffold. Adapted with permission from [53] 2024, Elsevier.

In the cases of Li et al. [63] and Carluccio et al. [53], two durations of time are indicated; the first time is for the electrochemical test, and the second time is for the immersion test. Carluccio et al. [52] compared the corrosion behavior of LPBF pure dense iron and cast iron, and they also worked with Fe35Mn scaffolds obtained by LPBF [53]. Figure 11a represents the equivalent circuit proposed by Carluccio et al. for the LPBF pure dense iron and cast iron. In the equivalent model proposed, *Rs* represents the resistance of the electrolyte, *CPEdl* represents the double layer capacitance, and Rp the polarization resistance. A constant phase element (CPE) was used to represent the non-ideal dielectric behavior of the working electrode due to the possible presence of a non-homogenous surface coating, impurities, dislocations, and grain boundaries. In Table 6, lower values of current density and corrosion rate and higher polarization resistance (Rp) are shown for the cast iron samples compared to the values obtained for the pure dense iron samples fabricated by LPBF, indicating slightly higher corrosion resistance of the cast iron samples compared to that obtained by LPBF.

Figure 11b represents the equivalent circuit for the Fe35Mn scaffold. The circuit consists of a Randles circuit to describe the reactions occurring at the metal–liquid interface where: *Rs* represents the resistance of the Hank’s solution, *CPEdl* represents the double layer capacitance, and *Rct* is the resistance to charge transfer. The secondary circuit represents the oxide layer and consists of *CPEox*, which is associated with the oxide layer capacitance; *Rox* is the resistance of the oxide layer; and *L* is associated with the inductive response of the oxide layer. The use of constant phase element (*CPE*) models is for non-ideal dielectric behavior possibly caused by non-uniform coatings, dislocations, impurities, and other defects. Comparing the corrosion rate from the immersion test of pure iron and Fe35Mn scaffolds, it is noted that Fe35Mn scaffolds present a higher corrosion rate (0.42 mm/year) than pure iron scaffolds (0.09 mm/year). Carluccio et al. associated all these differences with a combination of the effect of the manufacturing method, the effect of the addition of Mn, and the design of the scaffold, all of which can increase the corrosion rate [53].

**Table 6:** Degradation results of LPBF pure iron and its alloys.

Material	Part	Energy Density (J/mm^3^)	Corrosion Test	Conditions	CR (mm/year)	Icorr (mA/cm^2^)	Rp (Ω cm^2^)	Ref.
Cast Fe	-	-	Electrochemical test	OCP: 150 min; EIS: 10 mV, 100 kHz–10 mHz; Lp: ±0.25 V (vs. SCE), 0.166 mVs^−1^. In HBSS.	0.047 ± 0.003	4.05 ± 0.3	1410	[52]
Cold-Rolled Iron			Electrochemical test	OCP: 60 min; EIS: 10 mV; 100 kHz–10 mHz; Lp: −0.3–+0.5 V (vs. SCE),0.5 mVs^−1^. In r-SBF.	0.10 ± 0.01	0.0086 ± 0.0009	-	[63]
Cold-Rolled Iron			Immersion test	Samples immersed for 28 days in r-SBF.			-	[63]
LPBF Pure Fe	Dense	67	Electrochemical test	OCP: 150 min; EIS: 10 mV, 100 kHz–10 mHz; Lp: ±0.25 V (vs. SCE), 0.166 mVs^−1^. In HBSS.	0.072 ± 0.001	6.2 ± 0.1	1035	[52]
LPBF Pure Fe	Scaffold	10	Electrochemical test	EIS: 10 Mv, 100 kHz–10 mHz. In SBF.	-	0.00738 ± 0.00321		[55]
LPBF Pure Fe	Scaffold	10	Immersion test	Samples immersed for 30 days in SBF with a pH of 7.4	0.09 ± 0.02			[55]
LPBF Pure Fe	Scaffold	-	Electrochemical test	OCP: 60 min; EIS:10 mV; 100 kHz–10 mHz; Lp: −0.3–+0.5 V (vs. SCE), 0.5 mVs^−1^. In r-SBF.	1.18 ± 0.22	0.1028 ± 0.0192	-	[63]
LPBF Pure Fe	Scaffold	-	Immersion test	Samples immersed for 28 days in r-SBF	0.03		-	[63]
LPBF Pure Fe	Scaffold S0.2	-	Immersion test	Samples immersed for 28 days in r-SBF	0.19446	-	-	[60]
LPBF Pure Fe	Scaffold (Dense-in)	-	Immersion test	Samples immersed for 28 days in r-SBF	0.1389	-	-	[60]
LPBF Pure Fe	Scaffold (Dense-out)	-	Immersion test	Samples immersed for 28 days in r-SBF	0.17131	-	-	[60]
SLM Pure Fe	Scaffold S0.4	-	Immersion test	Samples immersed for 28 days in r-SBF	0.12501	-	-	[60]
LPBF Pure Fe	Scaffold		Electrochemical test	OCP: 60 min; Lp: −0.2–+0.5 V (vs. SCE),0.1 mVs^−1^. In HBSS.	0.049	0.0042		[64]
LPBF Fe25Mn	Scaffold	10	Electrochemical test	EIS: 10 mV,100 kHz–10 mHz. In SBF.	-	0.05125 ± 0.00752		[55]
LPBF Fe25Mn	Scaffold	10	Immersion test	Samples immersed for 30 days in SBF with a pH of 7.4	0.23 ± 0.05			[55]
LPBF Fe30Mn	Scaffold		Electrochemical test	OCP: 60 min; Lp: −0.2–+0.5 V (vs. SCE),0.1 mVs^−1^. In HBSS.	0.142	0.01191		[64]
LPBF Fe35Mn	Scaffold	62	Electrochemical test	OCP: 150 min; EIS: 10 mV,100 kHz–10 mHz; Lp: ±0.25 V (vs. SCE),0.166 mVs^−1^. In HBSS.	0.8			[53]
LPBF Fe35Mn	Scaffold	62	Immersion test	Samples immersed for 28 days in HBSS with a pH of 7.4	0.42 ± 0.03			[53]

Shuai et al. [55] compared the corrosion behavior of pure iron and Fe25Mn in scaffold form. Two tests were carried out: an electrochemical test and an immersion test. The results of electrochemical test showed that the pure Fe scaffold had less electronegative open circuit corrosion potential (−0.61 ± 0.04 V) and smaller corrosion current density (7.38 ± 3.21 μA cm^−2^) than the Fe25Mn scaffold (−0.74 ± 0.06 V and 51.25 ± 7.52 μA cm^−2^, respectively). More electronegative corrosion potential indicated a weakened corrosion resistance of the surface, while a higher corrosion current density revealed an accelerated corrosion rate. These results indicated the lower stability of Fe25Mn in SBF solution. The immersion test presented similar results as Fe scaffolds presented a lower corrosion rate (0.09 mm/year) than the Fe25Mn scaffold (0.23 mm/year). Authors explained this result due to the potential difference between different phases formed in the Fe25Mn, which led to galvanic corrosion, thus accelerating the corrosion rate.

Li et al. [63] performed electrochemical and immersion tests of pure iron scaffolds, and the results were compared with cold-rolled iron. The electrochemical results showed that LPBF iron exhibited higher corrosion current densities (102.8 µA/cm^2^) compared to cold-rolled iron (8.6 µA/cm^2^); also, a significantly higher corrosion rate was obtained for the LPBF iron sample compared to the cold-rolled iron sample (1.18 and 0.1 mm/year, respectively). From the immersion test, the corrosion rate of the scaffolds was 0.03 mm/year. They suggested that this slow corrosion rate of the LPBF iron scaffold in the immersion test is due to different corrosion mechanisms operating from the periphery to the center of the scaffolds. At the periphery, degradation took place quite fast, as evidenced by the formation of a brownish layer, and in the center of the scaffolds, there were no brownish corrosion products even after 28 days, so they observed that r-SBF attacked the struts on the periphery of the samples and exposed the grains on their surfaces, so the struts in the center of the samples stayed almost intact. Another reason for the low weight loss measured here may be related to the limitations of the weight loss measurement and sample cleaning. In the case of highly porous scaffolds, however, it is difficult to remove all the corrosion products, especially those in the center of the scaffolds.

Li et al. [60] also studied the degradation behavior of functionally graded porous iron. They carried out dynamic biodegradation tests for 28 days in a custom-built bioreactor in r-SBF at a flow rate of 0.3 mL/min to mimic moderate physiological fluid movement in bone. Four types of scaffolds were studied and are represented in Figure 9. The results showed that the specimens of the S0.2 group exhibited a weight loss of 16.7% (i.e., a corrosion rate above 0.194 mm/year), which was the highest among all the experimental groups, and the specimens of the group S0.4 had the lowest values of weight loss, 5.1% (above 0.125 mm/year). In the case of Dense-in and Dense-out groups, the Dense-out specimens showed a somewhat higher value of weight loss, 10.3% (above 0.1713 mm/year), than the Dense-in specimens, 8.9% (above 0.1389 mm/year). They also determined in this study that different biodegradation rates were found in the center of the AM porous iron specimens with and without functional gradients, even though they had the same local geometry. Their results suggest that topological design and functional gradients can be used as an important tool for adjusting the biodegradation behavior of AM porous metallic biomaterials.

Nie et al. [64] investigated the degradation behavior of pure iron and Fe30Mn by electrochemical tests in simulated physiological environments. As shown in Table 6, the corrosion current of the Fe30Mn scaffold (11.91 μA/cm^2^) was higher than that of the iron scaffold (4.20 μA/cm^2^), so the corrosion rate of the solid Fe30Mn sample (0.142 mm/year) was higher than that of the solid Fe sample (0.049 mm/year).

In Figure 10 it can be observed that dense parts fabricated by additive manufacturing present similar corrosion rates as iron parts fabricated by conventional methods. In general, scaffolds show a higher corrosion rate than the dense parts, and it has been studied by two types of assays: electrochemical and immersion tests. The electrochemical test seems to obtain a higher corrosion rate than the immersion test for the same material and the same scaffold design. The authors attribute this difference to the difficulty of thoroughly cleaning the scaffold and removing all the corrosion products generated during immersion in the simulated body fluid. Also, Figure 10 shows that, in general, iron alloy scaffolds show a higher corrosion rate than pure iron scaffolds. For FeMn alloys, results indicated that the electrochemical stability of the Fe matrix decreased after alloying with Mn, which could be deduced from the obtained electrochemical parameters as the higher current densities or the more electronegative corrosion potential values for the Fe alloys [55].

Using LPBF, a hierarchical porous architecture can be achieved, which increases the surface area of the metal exposed to the environment and, consequently, raises the degradation rate. Furthermore, the high cooling rates and the complex heat inputs, typical of LPBF, impart high levels of internal defects, dislocations, and residual stresses into the scaffold. This can cause local destabilization of the passive film, creating small cathodic sites resulting in localized corrosion and thus an overall increased corrosion rate. Previous studies have shown that additions of Mn can further change the dielectric behavior of the oxide layer due to the structural mismatch between the Fe and Mn oxide films, allowing for greater transport through the oxide layer and thus increasing the surface corrosion rate [53,65].

### 2.5. In Vitro Cytocompatibility

Iron is indispensable for life because it plays a crucial role in a wide range of vital biochemical activities. These activities include oxygen sensing, transport, short-term oxygen storage, catalysis, electron transfer, and energy generation [66]. There are limited excretion pathways for the iron ions released from a bioabsorbable implant. However, when the body’s optimal iron levels are exceeded, the body can self-regulate. Most of the absorbed iron will reach plasma, while the protein ferritin provides safe storage of the iron fraction retained by the cell. When the lifespan of the erythrocyte ends, it is shed through the gastrointestinal tract together with any remaining stored iron. This represents a significant mode of iron release and loss from the body. Other mechanisms of iron release involve sweating, bleeding, and excretion through the epidermis. These alternative mechanisms are important as there are no regulated processes for iron excretion through the liver or kidney in humans. In the case of biodegradable materials, there is a need to consider the biocompatibility not only of iron implants but also their corrosion products [62]. Table 7 summarizes the in vitro cytocompatibility tests of iron and FeMn alloys.

**Table 7:** In vitro tests of Fe and FeMn alloy scaffolds.

Material	Cell Assay	Cell Line	Duration	Results	Ref.
Fe	Indirect	MG-63	3 days	good cytocompatibility, with cells growing normally on the scaffolds	[55]
Fe25Mn	Indirect	MG-63	3 days	good cytocompatibility, with cells growing normally on the scaffolds	[55]
Fe30Mn	Indirect; cell proliferation	MC3T3-E1	7 days	RGR grade 0. The extract is not cytotoxic.	[64]
Fe30Mn	Direct; live/dead test	MC3T3-E1	7 days	seven days co-culture results in many live cells and only a few dead cells	[64]
Fe35Mn	Indirect; cell proliferation and MTT tests	MC3T3-E1	3 days	the scaffold displayed biocompatibility, high viability towards mammalian cells, and filopodia on the scaffold indicated that the alloy is suitable for osteoblast adhesion	[53]
Fe	Indirect; cell proliferation	MG-63	3 days	MG-63 viability in extended, long-term extracts (72 h) of iron specimens dropped to below 50%.	[63]
Fe	Direct; live/dead test	MG-63	1 day	revealed substantial and almost instant cytotoxicity	[63]

In vitro tests were carried out on scaffolds based on iron alloys. Two cell lines have been studied, the MG-63 osteoblastic cell line [55,63] and the MC3T3-E1 osteoblastic cell line [53,64], for direct and indirect cytocompatibility tests. For the indirect test, different time periods were studied (3 and 7 days) [53,55,63,64]. In general, all results show good cytocompatibility and no cytotoxic response. For direct assays, only two studies were found: for Fe for 1 day [63] and for Fe30Mn for 7 days [64]. For pure Fe, the direct test reported substantial and almost instant cytotoxicity in static cell culture [63]. For Fe30Mn scaffolds, results showed many live cells and only a few dead cells in contact with the sample. Moreover, live cells exhibited fusiform or polygonal shapes, indicative of proper cell adhesion [64].

Studies such as cell proliferation [53,63,64] and life/dead [63,64] assays are commonly used to measure the cytotoxicity of the material under investigation. Only Carluccio et al. used MTT to conduct these studies [53]. They utilized the cell proliferation assay to quantitatively measure the rate and extent of cell growth over time, providing insights into the capacity of the cells to divide and expand under the given experimental conditions. Additionally, they employed the MTT assay to assess cell viability, a critical parameter that reflects the metabolic activity of the cells and serves as an indicator of their overall health and functionality in response to the experimental material or treatment. Together, these assays provided complementary data on the effects of the tested material on both cell growth dynamics and cellular metabolic integrity.

### 2.6. In Vivo Studies

Table 8 shows the in vivo tests for iron-based alloys. Two materials were studied: Fe30Mn [64] and Fe35Mn [53]. Fe30Mn scaffolds were implanted in the lateral femoral condyle of rabbits for 48 weeks, and Fe35Mn scaffolds were implanted in the cranium of rats for 4 weeks. In both cases, the results showed good biocompatibility and osseointegration at 4 and 48 weeks. Nie et al. [64] also studied the mechanical properties of the Fe30Mn alloy, which is a critical parameter for osteosynthesis applications, because it is necessary that the implanted material provides continuous mechanical support during the healing process, including scaffold degradation and bone regeneration. The results of the mechanical properties showed continuous mechanical support during long-term degradation and dynamic bone remodeling.

**Table 8:** In vivo tests of FeMn alloys.

Material	Shape	Animal	Implantation Place	Duration	Results	Reference
Fe30Mn	Scaffold	Rabbit	lateral femoral condyle	48 weeks	biocompatibility and osseointegration performances in the repair of load-bearing bone defects	[64]
Fe35Mn	Scaffold	Rat	cranium	4 weeks	the implant integrated with the original bone, and even stimulated bone formation	[53]

All the biological characterization, both in vitro and in vivo, was performed for Fe and FeMn scaffolds manufactured by the LPBF process and not in dense parts. In the case of in vitro assays, different cellular lines were used, and direct and indirect tests were carried out (Table 7 and Table 8). In most cases, the results showed good cytocompatibility in in vitro tests [53,55,60,63,64]. Li et al. emphasized the importance of scaffold design since the results showed greater cell growth on scaffolds with a certain porosity [60]. In in vivo studies, only FeMn alloys were studied, and rats [53] and rabbits [64] were employed. The results showed good compatibility and good osseointegration.

The excellent biocompatibility of iron alloys and their high strength and ductility compared to magnesium and zinc alloys make iron one of the best potential materials for creating biodegradable metallic stents since the beginning of the 21st century. Due to its mechanical properties and workability, it could be used to create lighter and smaller scaffolds. The problem encountered by researchers working with iron is the slow corrosion time in in vivo conditions, which prevents the recognition of iron as fully biodegradable. The challenge will be to create new iron-based materials that, without losing their advantages, present a higher corrosion rate. For this purpose, research is carried out on alloy additions, the use of various manufacturing techniques to modify the structure and surface morphology of the material, or the use of additional materials as a coating to influence the course of corrosion [62]. Advancements in AM technologies now enable the direct fabrication of fully functional parts. LPBF offers the opportunity to customize implants at a lower cost due to its ability to produce parts with complex and intricate designs tailored to individual patients. [67].

## 3. Additive Manufacturing of Zn-Based Alloys

Zinc is most abundantly present in the human body in muscles and bones. It is essential for the regulatory function of several metalloenzymes, including alkaline phosphatase (ALP), and for the proper functioning of human cells [68]. Zn^2+^ ions play a significant role in bone metabolism by stimulating the growth and differentiation of human bone marrow mesenchymal stem cells (hMSCs) and inducing osteogenesis [69]. Furthermore, zinc contributes to endothelial cell metabolism and helps prevent physiological disorders in the inner walls of arteries, capillaries, and veins [70]. Given these physiological properties, Zn is being explored for cardiovascular and bone implants.

The biodegradation process of zinc is more appealing than that of magnesium (Mg) or iron (Fe). Zinc’s corrosion rate is intermediate—faster than Fe but slower than Mg. Unlike Mg, Zn does not generate hydrogen gas in physiological environments, and opposed to Fe, its biodegradation products do not accumulate and are biocompatible [71]. However, the mechanical properties of pure Zn are less attractive compared to Mg or Fe. Pure cast Zn has an ultimate tensile strength of approximately 30–40 MPa and exhibits non-uniform deformation, which are drawbacks for its use in medical devices [72]. Therefore, the main challenge in utilizing Zn lies in designing strategies to develop alloys with higher strength and uniform elongation.

The addition of alloying elements and thermomechanical refinement of the microstructure are the most common strategies to improve the mechanical performance of zinc. Alloying zinc with lithium (Li) or magnesium (Mg) improves its ultimate tensile strength. Adding elements such as silver (Ag), copper (Cu), iron (Fe), and manganese (Mn) enhances both uniform elongation and fracture elongation, while alloying with strontium (Sr) improves uniform elongation, and zirconium (Zr) increases fracture elongation. Based on mechanical properties, biodegradable zinc alloys can be categorized into three groups: high strength and low uniform elongation (ZnLi), medium strength and uniform elongation (ZnMg), and low strength and high elongation (ZnCu, ZnMn, and ZnAg) [72].

For applications in cardiovascular disease treatment or bone regeneration, specific mechanical properties are required. Uniform elongation is critical for stent applications, while high to medium strength is preferable for orthopedic implants. The in vivo biocompatibility of various Zn alloys has already been demonstrated. For instance, hot-extruded binary alloys such as ZnMg, ZnCa and ZnSr have shown biocompatibility and the ability to promote bone regeneration in animal studies [73]. Pure Zn wires have been proven biocompatible with arterial tissue, promoting regeneration [74]. Laser-cut ZnCu stents implanted in porcine coronary arteries have provided adequate structural support and suitable degradation rates [9].

The potential of zinc-based biodegradable implants is immense, particularly with the possibility of leveraging additive manufacturing techniques. This section focuses on the state of research into the additive manufacturing of zinc using laser powder bed fusion.

### 3.1. Linking Processing Parameters and Energy Density to Densification

The first study to explore the production of Zn parts using LPBF was presented in 2016 at the World Powder Metallurgy Congress and Exhibition (PM2016) by Lietaert et al. [75]. The research team from 3D Systems and KU Leuven utilized a customized ProX DMP 320 machine from 3D Systems (Rock Hill, SC, USA) featuring a 500 W laser and modifications to the assist gas flow. They began by determining a stable linear energy density (0.25 J/mm) through single-track (1D) scans. Subsequently, they optimized the track overlap and minimized smoke formation in 2D experiments by adjusting the hatch spacing, ultimately setting it at 100 µm. Further refinement was achieved in 3D experiments by setting the layer thickness to 30 µm, enabling the production of cubes with a relative density higher than 99.70%. Less than a year later, in 2017, Montani et al. [76] published a study investigating the processability of Zn using an unmodified LPBF machine. They produced porous structures with a relative density of 88%, attributing the high porosity to metal vaporization during manufacturing. This work highlighted the necessity of modifying the processing chamber to reduce porosity in the final parts. Since then, various studies have explored alternative strategies and parameters to achieve fully dense Zn parts.

**Figure 12:**
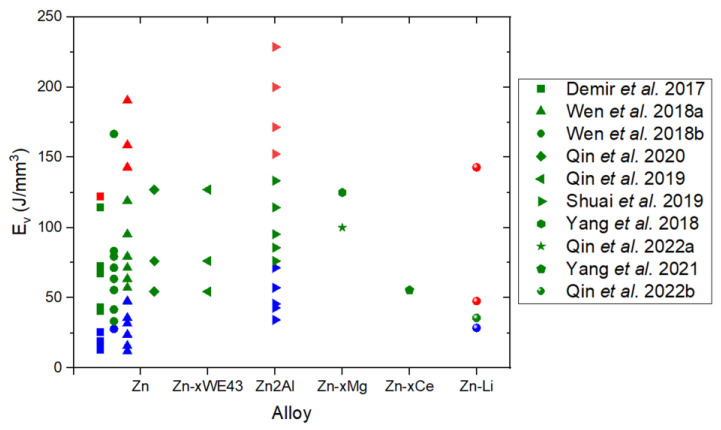
Volumetric energy densities (*E_v_*) used for fabricating Zn and Zn alloys parts by LPBF. Green indicates optimum conditions, red excess of energy, and blue lack of energy [33,77,78,79,80,81,82,83,84,85].

**Figure 13:**
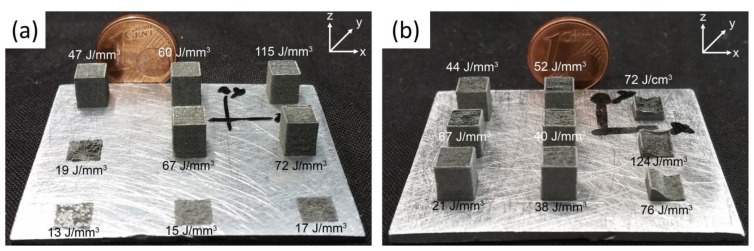
Appearance of LPBF-produced pure Zn parts showing the effect of fluence with (**a**) coarse particles and (**b**) fine particles Adapted with permission from [77]. 2024, Elsevier.

Figure 12 illustrates the volumetric energy density (*E_v_*) ranges explored by researchers printing Zn and Zn alloys via LPBF. Demir et al. [77] examined the influence of *E_v_* on the density of zinc parts using coarse (D_50_: 15 µm) and fine (D_50_: 9 µm) powders. As shown in Figure 13, samples printed with fine powders consistently exhibited porosity levels below 95%, and the process was unviable at energy values above 72 J/mm^3^. In contrast, high density parts (above 99%) were achieved with coarse powders working within an optimal *E_v_* range of 40–115 J/mm^3^, depicted as green squares in Figure 12. Red squares in the same figure indicate higher energy values, which resulted in parts with density below 95%, while blue squares represent insufficient energy levels that caused incomplete melting or missing parts. Demir et al. also investigated the impact of the processing atmosphere—specifically argon (Ar) and nitrogen (N_2_)—within the chamber, under gas jets, and in ambient air in an open chamber. They concluded that a stable processing environment was achieved using an open chamber with an Ar jet.

Wen et al. [78] investigated the effect of processing parameters on the densification of Zn samples using an LPBF machine (ACONITY, Herzogenrath, Germany) equipped with a specially designed gas circulation system to mitigate detrimental effects, such as Zn evaporation. Although they did not explicitly use the energy density (*E_v_)* to describe processing conditions, *E_v_* values were calculated based on input variables, including laser spot diameter (75 µm), layer thickness (30 µm), laser power (*P*: 60–120 W), hatch spacing (*h*: 60–120 µm) and scanning speed (*v*: 400–1200 mm/s). These values are shown as circles in Figure 12. A density above 99% was achieved for *E_v_* values ranging from 33 to 167 J/mm^3^ (green circles). Blue circles represent insufficient energy (*E_v_* < 30 J/mm^3^) resulting in a lack of fusion and density values below 95%. In further work, Wen et al. [33] explored the relationship between *E_v_* and sample density under varying laser powers and scanning speeds. By fixing the laser spot diameter (75 µm), layer thickness (30 µm), and hatch spacing (70 µm), they adjusted laser power and scanning speed to achieve *E_v_* values between 20 and 200 J/mm^3^. The optimal *E_v_* range for producing high-density parts with porosity below 0.5% was identified as 60–135 J/mm^3^, represented by green triangles in Figure 12. Blue triangles correspond to insufficient energy, causing a lack of fusion and irregular cavities, while red triangles indicate excessive energy, leading to Zn evaporation and gas entrapment.

Subsequent research on LPBF of Zn has leveraged Wen et al.’s [33] optimized *E_v_* range to fabricate high-density parts and scaffolds. For instance, Zn porous scaffolds were printed using *E_v_* = 95.2 J/mm^3^, achieving strut densities exceeding 99.8% as verified through industrial CT analysis [79]. Bulk Zn parts with relative densities above 99.5% have been produced by varying the scanning speed from 300 to 700 mm/s, corresponding to *E_v_* values of 54.4–126.9 J/mm^3^ (green diamonds in Figure 12).

**Figure 14:**
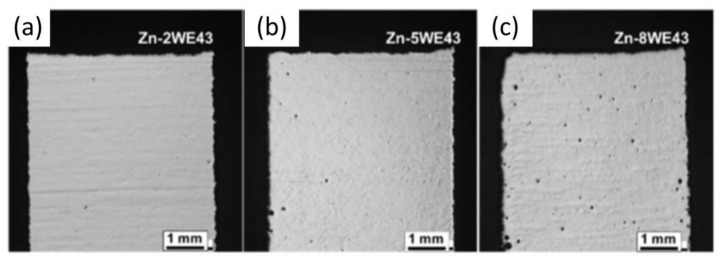
Picture of LPBF cross-sections of Zn-xWE43 bulk samples (**a**) Zn2WE43, (**b**) Zn5WE43 and (**c**) Zn8WE43 Adapted with permission from [80]. 2024, Elsevier.

Some studies on LPBF of Zn alloys have adapted the optimized parameters for pure Zn but often require preliminary adjustments to achieve high density. For example, Qin et al. [80] fabricated Zn-xWE43 (x = 2, 5, 8 wt%) bulk samples and porous scaffolds using processing parameters developed for pure Zn. Their optimal *E_v_* range (54.4–126.9 J/mm^3^) is indicated by left-pointing triangles in Figure 12, with densities exceeding 99%. However, as shown in Figure 14, increasing the WE43 content to 8 wt% reduced relative density to below 99.5%, whereas pure Zn and Zn-2WE43 samples achieved densities above 99.9% [80].

Shuai et al. [81] investigated the fabrication of Zn-2Al parts by varying laser power (60–160 W) and scanning velocity (200–500 mm/s) to determine optimal processing parameters for high-density parts. The hatch spacing and layer thickness were fixed at 70 µm and 50 µm, respectively. The energy density (*E_v_*) values that stabilized the process and formed continuous, smooth tracks are represented as green right-pointing triangles in Figure 12, ranging from 76.19 J/mm^3^ to 133.33 J/mm^3^. Lower *E_v_* values (blue right-pointing triangles) caused defects, including lack of fusion (*E_v_* < 45.7 J/mm^3^) and the balling effect (*E_v_* between 57.14 and 71.42 J/mm^3^). Excessively high *E_v_* values (red right-pointing triangles, *E_v_* > 152.38 J/mm^3^) led to significant powder evaporation. Relative densities achieved within the optimal *E_v_* range varied from 80% (*E_v_* = 76.19 J/mm^3^) to 98.3% (*E_v_* = 114.28 J/mm^3^).

Yang et al. [82] printed Zn alloys containing 1, 2, and 3 wt% cerium (Ce), using *E_v_* = 55.55 J/mm^3^ with a laser power of 50 W, a scanning speed of 300 mm/s, a layer thickness of 50 µm, and a hatch spacing of 60 µm. All samples achieved densities exceeding 98%. Zinc alloys with magnesium (Mg) have been produced as both bulk parts [83] and porous scaffolds [84]. Yang et al. [83] fabricated Zn-xMg (x = 0, 1, 2, 3, 4 wt%) bulk parts using fine Zn and Mg powders with mean diameters of 5 µm and 10 µm, respectively. After conducting preliminary optimization studies, the parameters were fixed at *P* = 200 W, *v* = 200 mm/s, *h* = 80 µm, and *t* = 100 µm, resulting in *E_v_* = 125 J/mm^3^ (green hexagon in Figure 12). Under these conditions, the highest relative density (98.2%) was achieved with 3 wt% Mg, while Zn2Mg samples showed the lowest density (96.6%). Pure Zn had a relative density of 97.4%. [83]. Qin et al. [84] fabricated porous Zn-Mg scaffolds using pre-alloyed Zn-xMg (x = 1, 2, 5 wt.%) powders with mean sizes of 19.5–20.9 µm. They employed optimized parameters for pure Zn (*P* = 60 W, *v* = 400 mm/s, *h* = 50 µm, and *t* = 30 µm), achieving *E_v_* = 100 J/mm^3^ (green star in Figure 12). The struts exhibited relative densities exceeding 99.5%.

Qin et al. [85] studied processing optimization for additively manufactured Zn-0.7Li. By varying laser power (40–120 W) and scan speed (200–1000 mm/s), while setting layer thickness to 70 µm and hatch spacing to 20 µm, they identified a narrow processing window. The optimum condition, represented as a green sphere in Figure 12 (*E_v_* = 35.7 J/mm^3^), was achieved with *P* = 400 W and *v* = 800 mm/s, resulting in a relative density above 99.5%. Excessively high *E_v_* values (*E_v_* ≥ 47.6 J/mm^3^) caused pore defects due to excessive energy input, while insufficient energy (*E_v_* ≤ 28.6 J/mm^3^) prevented full melting. Samples collapsed when laser power exceeded 100 W.

Advancements in LPBF for zinc (Zn) and its alloys have demonstrated significant progress in optimizing processing parameters to achieve high-density parts and scaffolds. The studies reviewed underscore the critical role of energy density (*E_v_*) in balancing key factors such as fusion, porosity, and evaporation, with optimal *E_v_* ranges varying based on material composition and part geometry. Researchers have effectively tackled challenges like Zn evaporation and porosity through tailored machine configurations, refined gas circulation systems, and precise parameter adjustments. While pure Zn has achieved relative densities exceeding 99.5%, alloying with elements such as Mg, Al, and Li has required further parameter fine-tuning to ensure similar or improved densification. These findings not only establish a solid foundation for additive manufacturing of Zn-based materials but also highlight the need for ongoing research to expand applications in areas such as biomedical implants, where material properties and process reliability are paramount.

### 3.2. Influence of the Microstructure on Hardness

In laser additive manufacturing, processing parameters play a crucial role in determining the microstructure, densification, and overall quality of the final part. The microstructure, along with inherent material properties such as composition and alloying elements, defines the performance of the final part for specific applications. In particular, the mechanical properties and corrosion resistance are strongly influenced by the microstructure of the material.

The microstructure of laser additive manufactured Zn parts is characterized by a strong texture, consisting of columnar grains aligned along the build direction, spanning multiple layers. The average grain size in LPBF Zn parts is finer than in cast, extruded, or hot-rolled Zn parts [33]. For instance, cast Zn typically exhibits equiaxed grains with average sizes ranging from 100 μm to 1 mm, while the smallest average grain size in extruded and hot-rolled Zn can reach 20 μm [33]. In contrast, LPBF Zn parts exhibit columnar grains with reported average widths ranging from 5 μm [33] to 9 μm [79,82].

**Figure 15:**
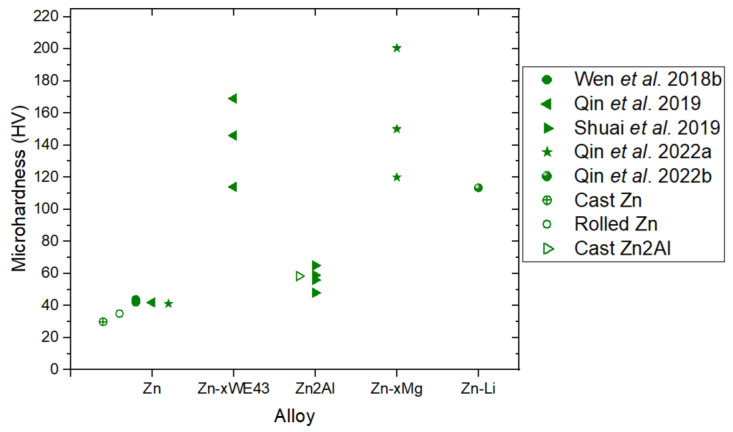
Microhardness of high-density Zn and Zn alloy parts manufactured by LPBF [78,80,81,84,85].

The finer grains produced during the laser additive manufacturing process result in higher microhardness values compared to rolled or cast Zn [77]. However, the degree of densification significantly influences the hardness of the parts. For example, Zn parts with relative densities below 95% show an average hardness of 30 HV, which is lower than that of rolled Zn (35 HV). Figure 15 illustrates the microhardness of high-density Zn and Zn alloy parts manufactured via LPBF, in comparison with cast and rolled Zn. High-density LPBF pure Zn parts (>99% relative density) achieve hardness values ranging from 40 HV to 45 HV, surpassing the microhardness of cast and rolled Zn [33,77].

The addition of alloying elements further enhances hardness through mechanisms such as the dilution of added elements in the Zn matrix, the precipitation of new phases, and the refinement of columnar grains. For instance, Shuai et al. [81] investigated the effect of laser energy density (*E_v_*) on the hardness of Zn2Al parts. Using *E_v_* values ranging from 76.19 to 133.33 J/mm^3^, they produced parts with varying grain size distributions and relative densities. The average grain size was coarser with increasing laser energy density, but also the parts were less porous. Figure 15 shows, using right-facing triangles, the values of the Zn2Al microhardness. Hardness increased from 48 HV to 64.5 HV with moderate energy input (76.2–114.3 J/mm^3^) due to the densification of the parts. Higher energy input (133.3 J/mm^3^) reduces the hardness to 56.5 HV. Zn-2Al parts manufactured with a low *E_v_* (76.2 J/mm^3^) exhibited the smallest grain size (2.21 μm), but also the lowest densification, reaching lower hardness than the cast alloy. At the highest *E_v_* (133.3 J/mm^3^), the densification is optimum, but the grain size is bigger (6.62 μm), reducing the hardness of the alloy.

Considerable increments in hardness are achieved by alloying Zn with lithium (Li) and magnesium (Mg). For instance, alloying Zn with 0.7% of Li increases the hardness to 113.4 HV (represented as a sphere in Figure 15) [85]. Hardness values exceeding 100 HV can also be achieved by alloying Zn with WE43 [80]. Figure 15 displays the microhardness of Zn-xWE43 alloys (indicated by left-facing triangles), with hardness ranging from 114 HV for a WE43 content of 2 wt.% to 170 HV for a WE43 content of 8 wt.%. Qin et al. [84] reported hardness values ranging from 120 to 200.4 HV by increasing the Mg content from 1 wt% to 5 wt% (depicted as stars in Figure 15).

Densification is a key factor influencing hardness, with high-density parts (>99% relative density) exhibiting superior hardness values. Additionally, the incorporation of alloying elements such as lithium, magnesium, and aluminum further enhances the hardness of Zn parts through grain refinement and phase precipitation. This highlights the importance of carefully optimizing both processing parameters and alloy compositions to achieve desired mechanical properties and performance in Zn parts for specific applications.

### 3.3. Mechanical Properties of Dense Structures and Scaffolds

In addition to hardness, tensile performance can also be enhanced by adjusting processing parameters and adding alloying elements. Table 9 summarizes the tensile mechanical properties of LPBF dense parts made of Zn and Zn alloys. Alloying Zn with WE43 significantly increases the yield strength and ultimate tensile strength (UTS) of the printed part but reduces its elongation [80]. In contrast, Yang et al. [83] improved the elongation of Zn by alloying it with Mg, achieving values as high as 7.2%. Yield strength and UTS were also considerably increased by adding 1, 2, 3, and 4 wt.% of Mg, with the best results obtained for the Zn3Mg alloy, as shown in Table 9. Alloying with Ce also enhanced the elongation of Zn to 7.5%, and the yield strength and UTS reached 180 MPa and 247 MPa, respectively, with a 2% Ce content [82]. Shuai et al. [81] studied the effect of laser energy density on the tensile properties of Zn2Al samples. Increasing the energy density (*E_v_*) up to 114.28 J/mm^3^ resulted in less porous samples with higher yield strength, UTS, and elongation. However, further increases in energy input led to a decrease in strength and elongation of the Zn alloy.

Tensile properties of LPBF Zn parts vary depending on processing parameters and fabrication strategy. As shown in Table 9, the ultimate tensile strength of pure Zn varies from 61.3 MPa [83] to 134 MPa [80], while the elongation of Zn ranges from 1.7% [83] to 12% [86].

The mechanical properties of Zn depend on the building direction. The anisotropy of LPBF Zn parts has been studied by Qin et al. [87] and Lietaert et al. [86], who examined the tensile properties of samples manufactured in two directions: parallel to the building direction (vertical) and perpendicular to the building direction (horizontal). Both studies found that vertically built samples exhibited higher strength than horizontally built ones. Qin et al. further investigated the relationship between scanning speed and the yield strength, UTS, and ductility of the samples [87]. For vertical samples, increasing the scanning speed strengthened the sample and increased its ductility. For horizontal samples, increasing scanning speed improved ductility, while strength remained similar (see Table 9).

**Table 9:** Tensile mechanical properties of LPBF dense parts made of Zn and Zn alloys.

Material	Ev (J/mm^3^)	Yield Strength (MPa)	UTS (MPa)	Elongation (%)	Ref.
Zn	66.7		134	10	[80]
Zn2WE43			298.5	1.8	
Zn5WE43			335.4	1	
Zn8WE43			154.1	0.9	
Zn	125	43.2	61.3	1.7	[83]
Zn1Mg		74	126	3.6	
Zn2Mg		117	162	4.1	
Zn3Mg		152	222	7.2	
Zn4Mg		132	166	3.1	
Zn2Al	76.19	120	170	9	[81]
	95.24	135	185	10	
	114.28	140	190	12	
	133.33	138	188	11	
Zn	55.55	79.9	103.6	5.10	[82]
Zn1Ce		140	210	6	
Zn2Ce		180.6	247.4	7.5	
Zn3Ce		182	230	6.8	
Zn (Vertical)	127(300 mm/s)	94	119	2.6	[87]
	76.19 (500 mm/s)	108	130	8	
	54.42(700 mm/s)	110.3	132	7	
Zn (Horizontal)	127(300 mm/s)	72	90	2.5	
	54.42 (700 mm/s)	75	92.3	5	
Zn (Vertical)	39	78	100	10	[86]
Zn (Horizontal)		55	79	12	

**Figure 16:**
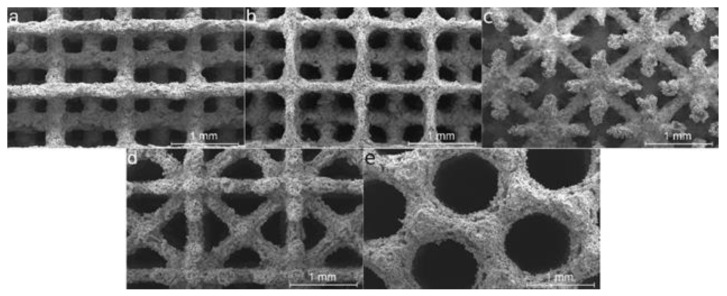
Scanning electron microscopy top view for scaffolds with (**a**) diamond unit cell, (**b**) dodecahedron unit cell, (**c**) octet truss unit cell, (**d**) FCC unit cell, and (**e**) 3D Kagome unit cell Reprinted with permission from [86]. 2024, Elsevier.

The mechanical properties of lattice-shaped Zn samples depend on several factors, in addition to processing parameters and alloying elements. Structural porosity, pore size, and geometry play a major role in the compressive performance of the scaffold. Table 10 shows the compressive mechanical properties of Zn and Zn alloy scaffolds fabricated by LPBF. Li et al. [60] studied the effect of the topological design of Zn scaffolds on their mechanical performance under compression. Samples with the lowest structural porosity and pore size exhibited the highest yield strength and elastic modulus. The selection of an appropriate unit cell is crucial for the mechanical properties of porous LPBF Zn parts. Figure 16 shows scanning electron microscopy images of Zn scaffolds with different unit cells. According to Lietaert et al. [86], scaffolds with Kagome unit cells exhibited the highest compressive strength. The elastic modulus, ultimate compressive strength, and yield strength increased with the alloying content in ZnxWE43 scaffolds [80]. However, in ZnxMg scaffolds, higher Mg content resulted in lower strength and modulus [84]. The compressive strength and modulus of Zn0.7Li scaffolds, with 80% structural porosity and pores larger than 800 μm, reached 18.2 MPa and 298 MPa, respectively [85].

**Table 10:** Compressive mechanical properties of LPBF scaffolds made of Zn and Zn alloys.

Material	Ev (J/mm^3^)	Structural Porosity/Pore Size	Geometry	Ultimate Compressive Strength (MPa)	Yield Strength (MPa)	Elastic Modulus (GPa)	Ref.
Zn	39	73%/700 μm	Diamond		4	0.4	[60]
		69%/Graded pore size 600–800 μm	Diamond		6	0.5	
		62%/600 μm	Diamond		11	0.8	
Zn	39	20–40%	Diamond	7–15 *			[86]
		22–40%	Dodecahedron	8–25 *			
		25–45%	FCC	10–50 *			
		22–35%	Kagome	15–50 *			
		30–50%	Octet Truss	9–30 *			
Zn	66.7	45%/600 μm	Diamond	23	13	0.95	[80]
Zn2WE43				60	51	1.91	
Zn5WE43				73	66	2.48	
Zn8WE43				51	51	2.54	
Zn	100	50%	Diamond				[84]
Zn1Mg				40		1.2	
Zn2Mg				35		1.3	
Zn5Mg				24		1	
Zn0.7Li	35.7	80%/820 μm	Gyroid	18.2		0.298	[85]

* Values of Plateau stress.

The mechanical properties of LPBF Zn and Zn alloy parts, including hardness, tensile strength, and compressive performance, are significantly influenced by processing parameters, alloying elements, and the design of the part. Adjusting processing parameters such as energy density and scanning speed can improve tensile performance, with alloying elements like WE43, Mg, Ce, and Li further enhancing strength and elongation. The anisotropy of LPBF Zn parts also plays a key role, with vertical samples generally exhibiting higher strength than horizontal ones. For lattice-shaped Zn samples, structural porosity, pore size, and unit cell design are crucial in determining compressive strength, with scaffolds featuring Kagome unit cells showing the highest compressive strength. Additionally, alloying Zn with elements such as WE43 increases the elastic modulus and strength, while excessive Mg content can lower the mechanical properties. Optimizing processing parameters and selecting appropriate alloying elements and structural designs are essential for achieving the desired mechanical properties in LPBF Zn and Zn alloy parts.

### 3.4. Corrosion Behavior

Corrosion behavior is a key factor in determining the suitability of a metal as a base material for manufacturing biodegradable implants. In the literature, this behavior is commonly evaluated through electrochemical tests and immersion studies. Among resorbable metals, the corrosion rate of Zn and its alloys is particularly promising for biodegradable implants, as it is generally higher than that of Fe (0.08 mm/year) but lower than that of Mg (1.58 mm/year) [81]. While alloying elements are intentionally added to Fe to modify its corrosion rate, alloying elements in Zn are not primarily intended to alter its degradation rate. Nevertheless, they influence its corrosion behavior, making it worthwhile to study the degradation rates of Zn alloys. Table 11 presents data from corrosion studies of additively manufactured Zn alloys, including results from both electrochemical and immersion tests. Corrosion rate values marked with an asterisk (*) have been calculated using the following equations:

(4) $$C_{R}=K_{1}\frac{i_{corr}\;EW}{\rho \;}$$

(5) $$EW=\frac{1}{\sum \frac{n_{i}f_{i}}{W_{i}}}$$

where *C_R_* is the corrosion rate in mm/year, *K*_1_ is a constat parameter with value 3.27 × 10^−3^ mm·g/μA·cm·year, *i_corr_* is the value of the corrosion current density given in μA/cm^2^ reported in each reference, and ρ is the density of the alloy in g/cm^3^. The equivalent weight (EW) is calculated with the valence of each element in the alloy (*n_i_*), the mass fraction of each element in the alloy (*f_i_*), and the atomic weight of each element in the alloy (*W_i_*).

**Figure 17:**
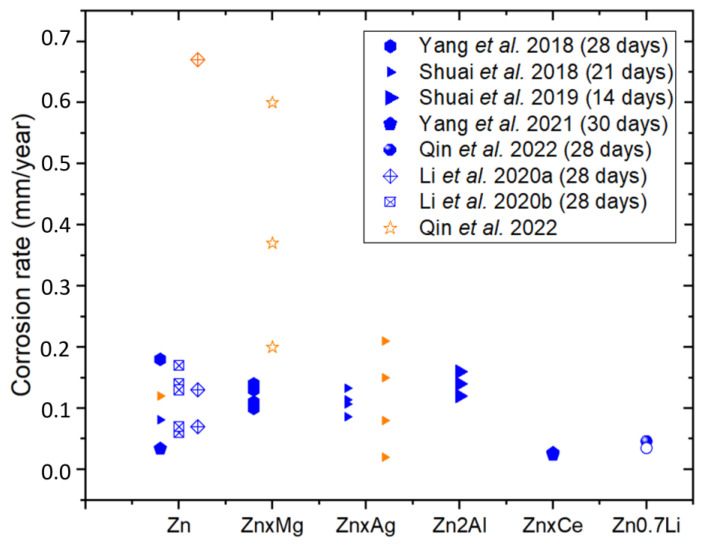
Corrosion rate of Zn and its alloys. In orange electrochemical tests, in blue immersion tests. Filled symbols correspond to dense parts and empty symbols correspond to scaffolds [81,82,83,84,85,88,90,91].

Figure 17 illustrates the corrosion rates from these studies in mm/year, obtained through electrochemical tests (orange) and immersion tests (blue). Results for dense parts and scaffolds are represented by filled and empty symbols, respectively. The corrosion rate of additively manufactured Zn and Zn alloy dense samples ranges from 0.02 mm/year to 0.21 mm/year. For scaffolds, some studies report corrosion rates similar to those of dense parts, while others indicate higher rates, around 0.7 mm/year. The required degradation rate for a biodegradable implant must align with the healing time of human bone. Since hard tissues take between 3 months and 1 year to repair, some authors suggest that the degradation rate of a resorbable implant should be slower than 0.5 mm/year [83], while others propose a suitable degradation rate of 0.2 mm/year [81]. The degradation rate is influenced by the type of test performed (electrochemical or immersion), alloying elements in Zn, processing parameters such as energy density, sample porosity, and surface roughness.

It is crucial to recognize that corrosion rate (CR) or *i_corr_* values cannot be directly compared when obtained using different electrolytes or scan rates, as these parameters significantly influence the results. Degradation studies of Zn alloys have used electrolytes such as simulated body fluid (SBF) [81,82,83,88], Hank’s solution [84,85,89], and r-SBF [90,91], each representing a distinct environment and contributing to differences in ion interactions and the formation of protective corrosion products.

Scan rates during electrochemical tests also play a critical role in CR measurements. Faster scan rates, such as 0.5 mV/s, can overestimate corrosion rates by not allowing sufficient time for protective layers to form and stabilize. This effect is evident in Zn0.7Li [85] and scaffold samples [91], where CR values are significantly higher at elevated scan rates. Conversely, slower scan rates or static immersion tests allow for the stabilization of corrosion products on the material’s surface, yielding more representative results. These observations emphasize the importance of standardized testing conditions when evaluating and comparing the corrosion behavior of Zn alloys.

Yang et al. [83] conducted electrochemical and immersion tests on dense ZnxMg parts, confirming that alloying Zn with Mg improves the corrosion resistance of this metal. Current densities decreased from 9.24 μA/cm^2^ to 3.62 μA/cm^2^, while corrosion rates determined by weight loss ranged from 0.18 ± 0.03 mm/year to 0.10 ± 0.02 mm/year. The Zn3Mg alloy exhibited the lowest current density and corrosion rate. The authors attributed this behavior to the increased grain boundary density caused by grain refinement due to Mg alloying or the formation of simonkolleite, which is more protective than the corrosion products of pure Zn, promoted by the release of Mg ions.

However, a different effect was observed in ZnxMg scaffolds. Qin et al. [84] found that higher Mg content increased the corrosion rate of Zn in scaffolds. This was attributed to the precipitation of Mg_2_Zn_11_ and MgZn_2_ phases, which have higher potentials than alpha-Zn, making galvanic corrosion the dominant corrosion mechanism for ZnxMg scaffolds.

In ZnxAg alloys, galvanic corrosion also accelerates Zn’s corrosion rate due to the presence of AgZn_3_ phase on the sample surface [88]. These results are true for immersion tests in SBF for 27 days. Electrochemical tests showed that corrosion current density decreases from 7.76 μA/cm^2^ in Zn to 1.47 μA/cm^2^ in Zn4Ag alloy, but higher Ag contents increased the *i_corr_* above the value for pure zinc. The divergences may be due to the differing timeframes of the two test methods.

**Figure 18:**
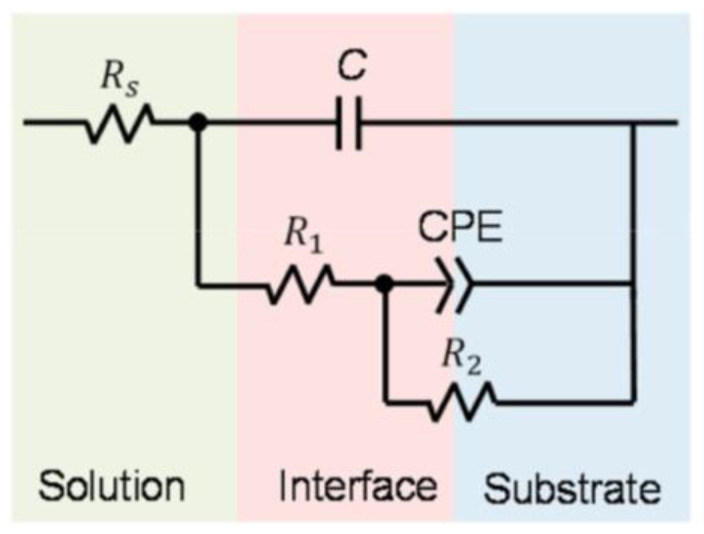
Equivalent electrical circuit for ZnxCe samples Reprinted with permission from [82]. 2024, Elsevier.

Yang et al. [82] demonstrated that alloying Zn with Ce improves corrosion resistance. The *i_corr_* decreased from 9 μA/cm^2^ in Zn to 6.97 μA/cm^2^ in the Zn3Ce alloy. The authors used an equivalent circuit (Figure 18) to quantitatively characterize the impedance spectra. R_s_ represented SBF resistance, R_1_ represented thin film resistance, and C described the capacitance of the corrosion product. R_2_ denoted charge transfer resistance, and CPE represented the constant phase element of the double layer at the Zn matrix–SBF interface. Results showed that R_1_ increased with Ce content, indicating that Ce promotes the formation of a protective film.

Shuai et al. studied the effect of volumetric energy density (*E_v_*) on the corrosion rate of Zn2Al alloys [81]. They conducted electrochemical and immersion tests in SBF, and results from both tests showed that the corrosion rate decreased as *E_v_* increased, attributed to improved densification of the dense parts with higher *E_v_*. Qin et al. [85] compared the corrosion rates of three different additively manufactured Zn0.7Li samples: bulk samples with as-built and polished surfaces and scaffolds with 80% porosity. The *i_corr_* of the sample with the as-built surface was approximately five times higher than that of the polished sample (101 ± 4.1 μA/cm^2^ vs. 28.5 ± 1.6 μA/cm^2^) and similar to that of the scaffold (111.2 ± 12.2 μA/cm^2^). Corrosion rates of porous samples, as calculated from immersion tests, were lower than those of dense samples with as-built surfaces. However, the mass loss of scaffolds was higher than that of dense samples. According to the authors, this discrepancy arose because the surface area of dense samples with as-built surfaces was underestimated, leading to an overestimation of the corrosion rate. The addition of Li improved Zn’s corrosion resistance by forming a passive layer of Li-rich corrosion products on the surface, which reduced the corrosion rate [89].

Li et al. investigated the corrosion rates of Zn scaffolds under dynamic and static conditions [90,91]. Static immersion degradation tests resulted in lower corrosion rates compared to dynamic immersion tests. Under static conditions, samples with varying porosity exhibited similar corrosion rates. However, under dynamic conditions, the porosity and topological design of scaffolds influenced the degradation rate, with higher porosity leading to increased corrosion rates [90].

The corrosion behavior of Zn and its alloys plays a critical role in determining their suitability for biodegradable implants. Factors such as alloying elements, processing parameters, surface properties, and porosity significantly influence degradation rates. Studies show that alloying Zn with elements like Mg, Ag, Ce, and Li alters its corrosion behavior, either enhancing its resistance or, in some cases, accelerating degradation due to galvanic effects. Processing parameters, such as volumetric energy density, improve densification and reduce corrosion rates, while surface finishing and scaffold designs introduce variability in behavior, particularly under dynamic conditions. Dynamic immersion tests generally reveal higher corrosion rates compared to static conditions, with porosity and topology playing crucial roles. This complex interplay of factors underscores the importance of optimizing composition, processing, and design to achieve desired degradation rates tailored to the healing times of human bone.

**Table 11:** Degradation results of additive manufactured Zn and Zn alloys.

Material	Part	Energy Density (J/mm^3^)	Electrochemical Test			Immersion Test		Ref
			Conditions	CR (mm/year)	icorr (μA/cm^2^)	Conditions	CR (mm/year)	
Zn	Dense	125	Samples soaked in SBF at 37 °C to obtain an OCP and polarization curves were recorded.	0.14 *	9.24 ± 1.21	Samples immersed in SBF at 37 °C for 4 weeks	0.18 ± 0.03	[83]
Zn1Mg				0.09 *	5.86 ± 1.42		0.14 ± 0.01	
Zn2Mg				0.07 *	4.63 ± 0.95		0.13 ± 0.03	
Zn3Mg				0.05 *	3.62 ± 0.76		0.10 ± 0.02	
Zn4Mg				0.06 *	3.71 ± 0.87		0.11 ± 0.04	
Zn	Dense		OCP: measured for 90 min (SBF). Scanning rate 1 mV/s	0.12	7.76	Samples immersed in SBF at 37 °C for 21 days	0.081	[88]
Zn2Ag				0.08	5.01		0.086	
Zn4Ag				0.02	1.47		0.107	
Zn6Ag				0.15	9.56		0.114	
Zn8Ag				0.21	13.94		0.133	
Zn2Al	Dense	95.24	Samples immersed in SBF at 37 °C. OCP ± 300 mV	0.18 *	11.75	Samples immersed in SBF at 37 °C for 14 days	0.16	[81]
		114.28		0.12 *	8		0.14	
		133.33		0.10 *	7.07		0.12	
Zn	Dense		OCP: 50 min, −200 mV to 200 mV at 0.05 mV/s. EIS: 10^−2^–10^6^ Hz, 10 mV (SBF)	0.13 *	9	Samples immersed in SBF for 30 days	0.034	[82]
Zn1Ce				0.12 *	8		0.027	
Zn2Ce				0.11 *	7.2		0.025	
Zn3Ce				0.10 *	6.9		0.024	
Zn0.7Li	Dense as built		EIS: frequency range 10^−2^–10^5^ Hz 10 mV (Hank’s solution)	1.5 *	101 ± 4.1	Samples immersed in Hank’s solution at 37 °C for 28 days		[85]
	Dense polished			0.43 *	28.5 ± 1.6		0.046	
	Scaffold (Porosity 80%)			1.6 *	111.2 ± 12.2		0.035	
Zn Scaffolds	Porosity 73%					Samples immersed in r-SBF for 28 days. Static and dynamic tests. Dynamic tests at a flow rate of 0.3 mL/min.	0.17 (Dynamic) 0.07 (Static)	[90]
	Porosity 69%						0.14 (Dynamic) 0.06 (Static)	
	Porosity 62%						0.13 (Dynamic) 0.07 (Static)	
Zn Scaffolds	Porosity 62%		r-SBF at 37 °C. Specimen was polarized from −0.2 V to +0.5 V potential versus OCP at 0.5 mV/s scan rate. For EIS: 1, 2, 7, 14, 21 and 28 days, 10 mV, 100 kHz	0.67 ± 0.04	45 ± 2	r-SBF at 37 °C for 28 days	0.13 (Dynamic) 0.07 (Static)	[91]
Zn	Scaffold (Porosity 50%)		Sample immersed in Hank’s solution for 1.5 h					[84]
Zn1Mg				0.20 *	13.5 ± 5.7			
Zn2Mg				0.37 *	24.9 ± 10.6			
Zn5Mg				0.60 *	40 ± 11.3			

* Values calculated with Equations (4) and (5).

### 3.5. In Vitro Cytocompatibility

The cytocompatibility of additively manufactured Zn and Zn alloys has been evaluated in in vitro conditions using direct and indirect tests with different cell lines. The available information on this topic is reported on Table 12. Commonly used cell lines include human osteosarcoma MG-63 cells, MC3T3-E1 pre-osteoblasts, and human telomerase reverse transcriptase mesenchymal stem cells (hTERT-MSCs). The preferred culture medium is Dulbecco’s Modified Eagle Medium (DMEM), supplemented with fetal bovine serum (FBS) and antibiotics such as penicillin and streptomycin. Cell viability depends on several factors, including the type of cytocompatibility evaluation test (direct or indirect), the alloying elements, the duration of the test, the culture medium, and the porosity of the material.

Several assays are employed to evaluate cytocompatibility comprehensively. The Live/Dead viability assay is commonly used to assess cell viability and cytotoxicity by staining cells with Calcein-AM and dead cells with Ethidium homodimer-1, enabling qualitative analysis through fluorescence microscopy [90,91]. Similarly, cytoskeletal staining techniques with DAPI and FITC-phalloidin reveal cellular morphology and attachment, observed via confocal microscopy [85]. These methods provide insights into the material’s effects on cellular structure, including the integrity of the cytoskeleton and nuclei.

Quantitative assays, such as the Cell Counting Kit-8 (CCK-8) and CellTiter 96^®^ assays, measure metabolic activity as an indicator of cell proliferation and viability. For instance, the CCK-8 assay involves exposing cells to Zn alloy extracts, with absorbance measurements at 450 nm reflecting cellular metabolic activity [81,82,83,92]. The CellTiter 96^®^ assay similarly uses extracts from Zn alloys to assess cell proliferation by detecting formazan products from metabolically active cells [90,91].

Additionally, specialized techniques like alkaline phosphatase (ALP) activity tests investigate early osteogenic differentiation in mesenchymal stem cells [92], while hemolysis tests evaluate compatibility with blood cells [92]. These complementary assays collectively provide a robust evaluation framework for the cytocompatibility of Zn alloys.

Studies have been conducted on both dense samples and scaffolds, with scaffolds generally showing better cell viability and adhesion than dense samples. Qin et al. [85] studied the cell adhesion of MC3T3-E1 pre-osteoblasts on Zn0.7Li bulk and scaffold samples. They observed poor cytocompatibility in dense samples, where cells shrank, assumed spherical morphology, and detached from the surface. However, cell adhesion was only monitored for 2 h, which is a short duration for comprehensive evaluation. In contrast, cells spread within scaffolds and exhibited healthier morphology with stretched pseudopods. Human osteoblast-like MG-63 cells remained viable and attached to the surface of Zn scaffolds after 24 h of culture in DMEM, with only a few cells showing compromised membrane integrity [90,91]. However, Zn scaffolds showed poor cytocompatibility in longer-term direct studies. Lietaert et al. reported that hTERT-MSC cells did not attach or grow on Zn scaffolds after 14 days in DMEM medium [86]. The differences in cell viability may also be due to varying test durations and cell types. Indirect tests using extracts generally show better cytocompatibility than direct tests, which typically report lower cell viability. However, extract concentration should be considered for a more accurate assessment of indirect cytocompatibility.

Li et al. [90] studied the cell viability of MG-63 cells cultured on Zn scaffolds and found that viability increased from 70% in direct tests to 95% in indirect tests using extracts prepared at 0.2 g Zn/mL in DMEM for 72 h. A similar 95% viability was observed in another study [91] for MG-63 cells cultured in Zn scaffold extracts for 24 and 48 h, although viability decreased to 85% after 72 h. Xia et al. found that 100% extracts from Zn scaffolds were cytotoxic to MC3T3-E1 cells [92], with a viability below 75% after 1 day. However, cell viability improved above 75% after 3 and 5 days, particularly when extracts were diluted to 50% and 10%.

**Figure 19:**
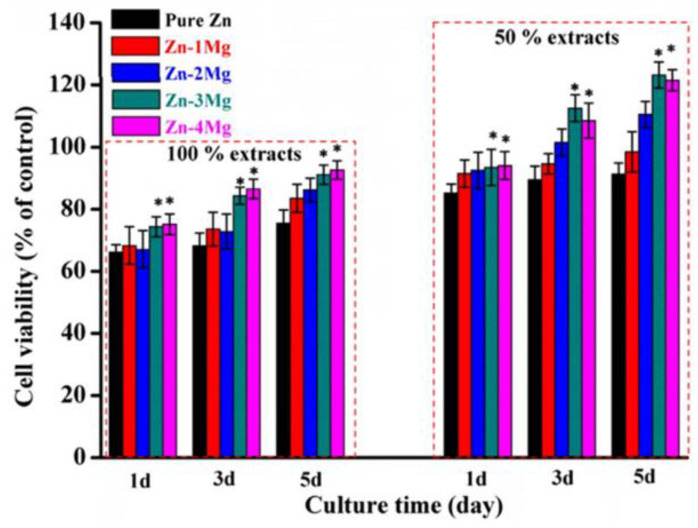
Quantitative viability results of MG-63 cells in extracts of LPBF processed ZnxMg. Data were normalized to the control group. Values were mean ±SD, n = 3, * *p* < 0.05 between the test group and the pure Zn group Reprinted with permission from [83]. 2024, Elsevier.

Yang et al. studied the effect of Mg content (0, 1, 2, 3, and 4) and extract concentration (100% and 50%) on the viability of MG-63 cells cultured in extracts from ZnxMg alloys [83]. Figure 19 shows the evolution of cell viability results for each extract concentration and each material with culture time. They found that Mg content increased cell viability, with pure Zn showing 91.3% viability after 5 days and Zn3Mg showing 123.2% viability. Cell viability increased with culture time and decreased extract concentration. The viability of MG-63 cells in 100% extracts after 1 day ranged from 65.5 to 78.3%, while in 50% extracts, viability increased to over 85% after 1 day and above 120% after 5 days. The presence of Mg in Zn not only proved non-cytotoxic but also promoted cell growth. In contrast, the addition of Ce in Zn had a slightly negative effect on cell viability. In indirect studies using bulk samples, the cell viability of MG-63 cells decreased slightly due to the presence of Ce in a Zn2Ce alloy compared to pure Zn [82], with Zn showing 83.75% viability and Zn2Ce showing 80.63%. Since cell viability was above 75%, the degradation products of Zn2Ce were not considered cytotoxic according to ISO 10993-5 standards [93]. Shuai et al. found that the presence of Al in additively manufactured Zn-dense parts negatively affected the viability of MG-63 cells [81]. Only 67.5% of cells cultured in 100% extracts from Zn2Al alloy remained viable after 1 day, and it was necessary to dilute the extracts to 50% to achieve over 80% viability after 1 day. Cell viability improved with longer culture durations.

The biocompatibility of Zn alloys for stent applications has been studied. Yang et al. performed hemolysis tests on LPBF Zn2Ce, using extracts prepared at a ratio of 1:3 mL/cm^2^ [82]. The tests were performed according to the ISO 10993-4:2002 standard [94]. In this study, human blood was exposed to the extracts obtained from each material. Results showed hemolysis percentages for Zn and Zn2Ce were lower than 5%, indicating good blood compatibility. Zn2Ce exhibited a hemolysis percentage of 3.82%, slightly higher than pure Zn (2.73%), indicating a minor reduction in blood compatibility due to the presence of Ce.

The cytocompatibility of additively manufactured Zn and Zn alloys varies depending on factors such as alloy composition, test methodology, and duration. Scaffolds generally exhibit better cell viability and adhesion compared to dense samples, likely due to their enhanced porosity and structure, which support cell attachment and growth. The presence of Mg in Zn alloys has been shown to promote cell viability and growth, while the inclusion of elements, such as Ce and Al, can negatively impact cytocompatibility, particularly in undiluted extract concentrations. Indirect cytotoxicity tests often yield more favorable results than direct tests, emphasizing the importance of test conditions and extract concentration in assessing biocompatibility. Hemolysis studies on Zn and Zn2Ce alloys confirm good blood compatibility, with hemolysis percentages well below the threshold for biocompatible materials. Overall, Zn alloys demonstrate potential as biomaterials, particularly in scaffold forms and with appropriate alloying and processing, although further research is needed to optimize their long-term biocompatibility.

**Table 12:** In vitro cytocompatibility tests of additive manufactured Zn and Zn alloys.

Material	Cell Assay	Cell Line	Duration	Medium	Conditions	Results	Ref.
ZnxMg	Indirect CCK-8	MG-63 cells	6 h, 1, 3, and 5 days	DMEM + FBS	Dense samples 100% and 50% extracts. (1.25 cm^2^/mL).	Good viability; better in 50% extracts. Mg content increases cell viability. Zn-3Mg best viability.	[83]
ZnxCe	Indirect CCK-8	MG-63	1, 3, and 7 days	DMEM + FBS, antibiotics	Dense samples. Extracts (1.25 cm^2^/mL).	Zn2Ce viability (80.6%), slightly lower than Zn (83.75%).	[82]
Zn0.7Li	Direct (cytoskeletal and nuclear staining)	MC3T3-E1	2 h	Cell suspension over the surface of samples.	Bulk and porous samples.	Better cell adhesion, viability and healthier morphology on porous samples.	[85]
Zn2Al	Indirect CCK-8	MG-63	1, 4, and 7 days	DMEM + FBS, antibiotics	Dense samples. 100% and 50% extracts. (1.25 cm2/mL).	Viability 67.5% (100% extracts); >80% (50% extracts). Viability increased over time.	[81]
Zn scaffolds	Direct live/dead	MG-63	24 h	DMEM + 1 g/L glucose + FBS		Most cells were viable (>70%)	[90]
	indirect CellTiter 96^®^		24, 48, and 72 h		Extract 0.2 g Zn/mL for 72 h	After 24 h, viability > 95% for all specimens	
Zn scaffolds	Direct Calcein staining and DNA measurement	hTERT-MSCs	14 days	high glucose DMEM + FBS + 1% PenStrep	Static seeding and dynamic seeding in a bioreactor	No cell attachment and growth for the Zn scaffolds.	[86]
Zn scaffolds	Indirect CCK-8 and ALP activity	MC3T3-E1	1, 3, and 5 days	α-MEM + 10% FBS	100%, 50%, and 10% extracts	Viability < 75% (100% extract afetr 1 day); viability > 75% after 3 and 5 days.	[92]
Zn scaffolds	Direct live/dead	MG-63	24 h	DMEM + 1 g/L glucose + 10% FBS		Most cells were viable, results similar to Ti6Al4V	[91]
	Indirect CellTiter 96^®^		24, 48, and 72 h		Extract 0.2 g Zn/mL for 72 h	Viability > 95% at 24, decreased to 85% at 72 h	

DMEM: Dulbecco’s Modified Eagle Medium. FBS: fetal bovine serum. hTERT-MSCs: human telomerase reverse transcriptase mesenchymal stem cells. α-MEM: minimal essential medium.

### 3.6. In Vivo Studies

In vitro results cannot be directly compared to the behavior of materials in vivo, as the degradation processes differ significantly between these two environments. A material that exhibits cytotoxicity in in vitro tests will not necessarily be cytotoxic when implanted in vivo [92]. A deeper understanding of a material’s biocompatibility is typically achieved through in vivo experiments. Despite the importance of such studies to assess the degradation process of implants in complex biological environments, research on the in vivo performance of additively manufactured Zn implants remains limited.

The osteogenic capacity and degradation behavior of Zn scaffolds fabricated by LPBF have been investigated in vivo by implanting scaffolds into predrilled defects in the femurs of New Zealand rabbits [83,92]. Table 13 summarizes these studies. Qin et al. [83] conducted a 12-week study, while Xia et al. [92] extended their observations to 24 weeks. Both studies reported the formation of fibrous connective tissue between the scaffolds and bone tissue as part of the host response, which initially impeded direct bone-scaffold contact [83]. Over time, the tissue became thinner, and by 24 weeks, new bone was integrated into the scaffolds [92]. The presence of Mg in Zn1Mg scaffolds improved osseointegration, with full integration into surrounding tissue achieved after just 12 weeks [83].

The in vivo performance of Zn stents fabricated by additive manufacturing has not yet been studied. However, investigations of Zn stents produced using other fabrication techniques provide insights. For example, Zhou et al. [9] implanted a Zn0.8Cu stent into porcine coronary arteries in a 2-year study. The stent demonstrated sufficient structural support, an appropriate degradation rate, and retained its integrity for up to 6 months. Furthermore, the corrosion process was uniform, without the accumulation of corrosion products.

**Table 13:** In vivo studies of additive manufactured Zn and Zn alloys.

Material	Shape	Animal	Implantation Site	Duration	Results	Ref.
Zn	Scaffold	Rabbit	Femur	24 weeks	Successful osseointegration of the scaffold	[92]
Zn and Zn1Mg	Scaffold	Rabbit	Femur	12 weeks	Osseointegration of Zn1Mg scaffolds. Fibrous connective tissue between bone tissue and Zn scaffold	[83]

In vivo studies are essential to comprehensively evaluate the biocompatibility and degradation behavior of Zn-based implants, as in vitro results cannot fully replicate complex biological environments. Although research on the in vivo performance of additively manufactured Zn implants is limited, existing studies demonstrate promising outcomes for Zn scaffolds, including osseointegration and controlled degradation over time. The presence of alloying elements like Mg further enhances scaffold integration. While the in vivo performance of additively manufactured Zn stents remains unexplored, studies on Zn stents fabricated by other techniques indicate their potential for adequate structural support, uniform corrosion, and favorable degradation rates, highlighting the need for further investigation into these innovative biomaterials.

## 4. Discussion

Iron (Fe) and zinc (Zn) alloys offer distinct advantages and limitations for biodegradable medical implants, particularly in bone and cardiovascular applications. Iron-based alloys excel in mechanical strength and structural support, making them ideal for applications like stents and high-load implants. However, their inherently low corrosion rate limits biodegradability, necessitating alloying or structural modifications. FeMn alloys, for instance, enhance both corrosion and mechanical properties but may introduce toxicity risks. In contrast, zinc-based alloys strike a balance between biodegradation rate and biocompatibility, with corrosion products that are naturally metabolized by the body. However, their lower mechanical strength compared to iron requires alloying with elements like magnesium or lithium to meet the demands of load-bearing applications.

### 4.1. Linking Processing Parameters and Energy Density to Densification

From a manufacturing perspective, Fe alloys present challenges due to their higher melting points, necessitating higher energy densities in laser powder bed fusion (LPBF) processes. Studies show that precise parameter optimization, such as balancing laser power and scanning speed, enables dense and defect-free parts. Zn alloys, on the other hand, have lower melting points, which reduces energy requirements but increases risks of vaporization and porosity. Innovations like optimized gas circulation systems have mitigated these challenges, enabling the fabrication of dense Zn components. However, the narrower processing windows for Zn alloys highlight the need for stringent control during manufacturing.

Conflicting knowledge regarding energy density ranges effects in the LPBF of Fe highlights the complexities of achieving optimal part quality. While Song et al. [51] reported high-density parts with energy densities as high as 2500 J/mm^3^, Shuai et al. [55] achieved similar results with energy densities as low as 20 J/mm^3^, and Carluccio et al. [52,53,54] with energy densities of 67 J/mm^3^. These variations likely result from differences in laser modes (CW vs. PW), power, and scanning speeds, compounded by divergent experimental setups and methodologies. Resolving these discrepancies requires systematic research to isolate and standardize key parameters such as laser power and scan speed.

In the LPBF of Zn, conflicting findings emerge primarily in energy density ranges and alloying content effects. Studies report varying optimal energy density ranges, with Demir et al. [77] suggesting 40–115 J/mm^3^, and Wen et al. [78] indicating 60–135 J/mm^3^ for high-density parts. Contradictions also arise regarding the effects of excessive energy densities, which some studies associate with porosity and evaporation while others consider beneficial for certain alloys. Conflicting preferences for processing atmospheres, such as open chambers with argon jets [77] versus specialized gas systems [78], complicate reproducibility. Alloying content further introduces variability, as higher percentages of elements like WE43 or Mg often reduce density despite following optimized parameters [80,83].

Further research is vital to address these knowledge gaps in LPBF of Fe and Zn. For Fe, a harmonized understanding of energy density ranges for pure Fe and alloys is necessary, with attention to the effects of CW and PW modes and a deeper exploration of the influence of energy density on densification. For Zn, comprehensive energy density mapping and standardizing processing atmospheres are essential to resolve disparities.

### 4.2. Influence of the Microstructure on Hardness

Microstructurally, Fe and FeMn alloys exhibit excellent hardness and strength due to grain refinement and the stabilization of austenitic phases. This refinement is especially pronounced in Fe35Mn, which combines small grain sizes with high hardness. Conversely, Zn alloys achieve superior hardness and strength through alloying elements like Mg and Al, which enhance grain refinement and phase precipitation. Fe alloys offer a hardness comparable to mild steel. Zn alloys have demonstrated increased microhardness in LPBF parts, making them competitive for applications requiring moderate mechanical strength and flexibility.

Research on LPBF of Fe and FeMn alloys reveals inconsistencies in observed microstructures, grain size trends, and the interplay between processing parameters and material behavior. While columnar grains are typically associated with LPBF processes, equiaxed grains have also been reported in pure Fe [52,54]. In FeMn alloys, microstructural variability is further influenced by Mn content, with Fe35Mn exhibiting columnar γ-grains [53,54] and Fe25Mn showing equiaxed grains with mixed phases [55]. Variations in grain size trends, like reductions observed at higher scanning speeds [51], add complexity to understanding how process parameters and composition interact, emphasizing the need for clarity in this area.

In LPBF of Zn, conflicting knowledge centers on the balance between grain size, densification, and hardness, particularly under varying laser energy densities. Finer grains tend to increase hardness but are associated with poor densification at low *E_v_*, while high *Ev* improves densification but coarsens grains, reducing hardness. These contradictory outcomes, especially in Zn2Al systems [81], highlight an unclear balance between densification and microstructural refinement. Alloying elements such as Li [85], Mg [84], and WE43 [80] demonstrate a broad range of effects enhancing hardness, potentially due to differences in compositional uniformity or processing conditions.

Future research should focus on resolving these inconsistencies and advancing understanding in key areas. For Fe, systematic studies on LPBF parameters, phase transformations, and mechanical properties are crucial to clarify the conditions that favor equiaxed versus columnar grains in pure Fe. For FeMn alloys, understanding the phase transformation pathways under varying Mn content and LPBF conditions is critical to elucidate how these factors influence phase stability and microstructure. Systematic investigations into the interplay between *E_v_*, densification, grain size, and hardness for both pure Zn and Zn alloys are crucial for optimizing processing parameters. The effects of alloying elements also demand deeper exploration, with comparative studies under controlled LPBF conditions needed to isolate the roles of composition and processing parameters.

### 4.3. Mechanical Properties of Dense Structures and Scaffolds

The mechanical properties of LPBF Fe and Zn exhibit distinct characteristics shaped by processing parameters, alloying, and design considerations. LPBF Fe demonstrates high yield strength and tensile properties, with dense parts achieving up to 421 MPa due to grain refinement and rapid cooling rates. Scaffold structures, however, show lower strength than dense parts, with properties influenced by porosity, strut thickness, and alloying with Mn. FeMn scaffolds outperform pure Fe in strength, with yield strengths up to 137 MPa, though no direct correlation between Mn content and strength is evident. These properties make Fe particularly suited for load-bearing biomedical applications. In comparison, LPBF Zn benefits from alloying with elements like Mg, Ce, and WE43, which improve yield strength, UTS, and elongation, though excessive Mg can reduce compressive performance. Zn exhibits anisotropy, with vertically built samples generally stronger than horizontal ones, and scaffold designs like Kagome unit cells achieving superior compressive strength. While Zn offers moderate strength and ductility, making it suitable for resorbable implants, its mechanical performance is more sensitive to structural and compositional optimizations compared to Fe.

Studies on the mechanical properties of LPBF Fe and its alloys reveal some discrepancies in the mechanical performance of scaffolds depending on their porosity and strut thickness. While thicker struts generally provide higher strength, functionally graded designs show intermediate performance, suggesting a complex relationship between design parameters and mechanical properties that remains poorly understood.

The mechanical properties of LPBF Zn and its alloys present some areas of conflict, particularly concerning tensile properties and the effects of alloying elements. For instance, ultimate tensile strength (UTS) values for pure Zn range widely between 61.3 MPa [83] and 134 MPa [80], and elongation varies from 1.7% [83] to 12% [86], reflecting inconsistencies likely due to differences in experimental protocols, processing parameters, and sample quality. The impact of Mg alloying is similarly contentious, with some studies reporting that Mg enhances UTS and elongation [83], while others show reduced strength and modulus at higher Mg concentrations [84]. This suggests that the effects of Mg are highly dependent on specific conditions, such as microstructure and defect levels. The influence of structural design, such as porosity and unit cell geometry, also yields mixed results, with findings showing that some designs achieve higher strength independently of porosity, while others suggest porosity plays a critical role, underscoring the need for deeper investigation.

Standardizing testing methodologies, including sample preparation and mechanical testing protocols, is essential to improve comparability across studies. Further studies on the relationship between porosity, pore size, and unit cell geometry in lattice structures require both experimental and computational analysis. For LPBF Zn, investigations into the mechanisms of Mg alloying are needed to understand its effects on tensile and compressive properties under various processing conditions.

### 4.4. Corrosion Behavior

Corrosion behavior marks a key distinction between the two material classes. Iron’s slow corrosion is a disadvantage for biodegradability, though alloying with Mn accelerates degradation while maintaining structural integrity. Scaffold designs further enhance corrosion rates due to increased surface area. Zn alloys, in contrast, degrade at rates more suitable for bone healing and vascular applications, with less risk of harmful accumulation. Alloying Zn with elements like Mg improves corrosion resistance and forms protective layers, but excessive Mg or Ag content can lead to galvanic corrosion. Porous scaffold designs amplify these effects, offering tailored degradation rates to match specific clinical needs.

In LPBF studies of Fe and its alloys, significant discrepancies hinder a unified understanding of corrosion behavior. One contentious area is the impact of LPBF manufacturing methods, with some studies linking increased corrosion rates in LPBF-fabricated iron to residual stresses and surface defects, while others report similar corrosion resistance compared to cast or cold-rolled iron [52,63]. Additionally, the influence of Mn on FeMn alloys is not well understood. While Mn addition is generally associated with increased corrosion rates, the extent of its effect varies across studies, attributed to differences in microstructure, oxide film properties, and galvanic interactions between alloy phases [53,55,64]. Lastly, scaffold design, including porosity and functional grading, adds variability to observed corrosion rates, with no consensus on the underlying causes.

Similar conflicts exist in the corrosion studies of LPBF Zn and its alloys, particularly concerning the effects of alloying elements and structural factors. For instance, while Mg alloying has been shown to improve corrosion resistance in dense Zn by forming protective products like simonkolleite [83], it accelerates corrosion in Zn scaffolds due to galvanic interactions between Mg2Zn11 and MgZn2 phases [84]. Ag alloying in ZnxAg alloys also presents inconsistencies, with immersion tests showing accelerated corrosion from galvanic effects of AgZn3 phases but electrochemical tests suggesting reduced corrosion at lower Ag contents [88]. Dynamic immersion testing highlights the significant impact of porosity and scaffold topology, which leads to higher corrosion rates compared to static conditions, though the exact interactions under physiological conditions remain unclear [90,91]. These conflicts emphasize the complex interplay of microstructure, alloying, and environmental factors in Zn corrosion behavior.

Addressing these inconsistencies requires standardizing corrosion testing protocols for both Fe and Zn with consistent parameters, such as electrolyte composition, temperature, scan rates, and test duration to improve comparability. For FeMn alloys, mechanistic studies should focus on Mn’s role in oxide layer properties and galvanic interactions. In Zn studies, deeper exploration of Mg and Ag alloying mechanisms, including phase formation and microstructural effects, is needed to resolve discrepancies in their impact on corrosion resistance. Research into scaffold design should address how porosity and topology influence ion exchange and degradation in dynamic biological environments. Accurate measurement of scaffold surface area and long-term studies on protective film evolution in both Fe and Zn alloys are also critical for predicting performance in real physiological conditions. These efforts will advance the understanding of LPBF materials and enable the design of better biodegradable implants.

### 4.5. In Vitro and In Vivo Studies

Biocompatibility studies further highlight the strengths and weaknesses of each material. Fe alloys show good in vivo performance, supporting bone remodeling and vascular integration. However, pure iron exhibits cytotoxicity in some in vitro assays, whereas FeMn alloys promote better cell adhesion. Zn alloys, on the other hand, show exceptional cytocompatibility and osteogenic potential, with promising in vivo results for osseointegration. The lower hemolysis and toxicity rates of Zn alloys further enhance their suitability for stents and scaffolds. Nevertheless, more research is needed to fully optimize both materials for their respective applications, ensuring consistent long-term performance.

The in vitro and in vivo behavior of LPBF Fe and its alloys reveals significant conflicting findings, particularly regarding cytotoxicity and biocompatibility. Pure Fe shows inconsistent cytotoxicity, with direct tests often indicating significant cytotoxic effects, while indirect tests suggest favorable outcomes, highlighting the impact of testing conditions such as material exposure and culture media. Mn alloying in FeMn alloys generally improves cytocompatibility compared to pure Fe, but variations in cell adhesion, proliferation, and live/dead assay results for different Mn concentrations, like Fe30Mn and Fe35Mn, create uncertainty about Mn’s precise role in biocompatibility. In vivo studies are limited in scope, often focusing on specific alloys like FeMn and restricted animal models, resulting in inconsistent findings regarding osseointegration and degradation mechanisms. Furthermore, scaffold design, including variations in porosity and topology, complicates biocompatibility assessments, as different designs yield conflicting results on cell growth and overall material performance.

The in vitro and in vivo behavior of LPBF Zn and its alloys also feature significant areas of conflict, especially concerning the influence of alloying elements and test conditions. Mg generally promotes cytocompatibility and osseointegration, but its effects in dense forms of Zn are inconsistent. Ce exhibits mixed impacts, with slight reductions in cell viability but no major cytotoxic effects, while Al negatively affects cytocompatibility in direct tests. Discrepancies between direct and indirect testing methods further complicate interpretations; indirect tests often show higher cell viability than direct tests, with results varying based on extract concentrations. Scaffold porosity usually enhances cell viability, but differences between static in vitro tests and dynamic in vivo conditions, such as fluid flow, create inconsistencies in observed performance. This highlights the challenges in correlating static lab-based tests with the dynamic, multifactorial environment of living tissues.

To resolve these conflicting findings, further research is required to standardize cytocompatibility testing protocols for both LPBF Fe and Zn, addressing variables like culture media, test duration, and extract concentration. For LPBF Fe, studies must focus on the mechanisms driving cytotoxicity in pure Fe, the role of ion release, and interactions with biological systems. For LPBF Zn, understanding the interplay of alloying elements, such as Ce and Al, and their long-term biological responses will help optimize alloy compositions. Finally, for both metals, further investigation into scaffold designs, including porosity and topology, under both static and dynamic conditions, will aid in balancing biocompatibility with mechanical stability and degradation rates for safer and more effective medical applications. Dynamic testing systems that mimic in vivo conditions are crucial for bridging the gap between in vitro and in vivo findings. Expanding in vivo research to include diverse alloy compositions and animal models will provide more comprehensive insights into long-term biodegradation, osseointegration, and ion release effects.

## 5. Conclusions

Iron-based and zinc-based alloys hold immense promise for biodegradable medical implants due to their unique properties and potential for customization through advanced manufacturing techniques like laser powder bed fusion (LPBF). Iron alloys, particularly FeMn variants, exhibit high mechanical strength and biocompatibility, making them suitable for cardiovascular and orthopedic applications. However, their slow corrosion rates pose challenges, prompting the use of alloying, microstructural refinement, and scaffold design to accelerate degradation and improve performance. Meanwhile, LPBF processing parameters critically influence material properties, as optimal energy density and alloy compositions are necessary to balance density, mechanical strength, and biodegradation rates. Advancements in LPBF have enabled the fabrication of dense iron and FeMn parts with customizable porosity, significantly enhancing their applicability in tissue scaffolds and stents.

Similarly, zinc and its alloys have emerged as excellent candidates for biodegradable implants due to their biocompatibility, intermediate corrosion rates, and favorable mechanical properties. LPBF techniques have been instrumental in overcoming the limitations of pure zinc, such as low tensile strength, by introducing alloying elements like magnesium and lithium. These alloys offer improved strength and controlled biodegradation, aligning with the healing timelines of bone and vascular tissues. However, challenges such as porosity, Zn evaporation, and localized corrosion persist, requiring meticulous optimization of processing parameters and scaffold designs. Both iron and zinc alloys demonstrate great potential for advancing the field of biodegradable implants, but further research is necessary to address biocompatibility, long-term performance, and clinical integration.

Further research is essential to address several knowledge gaps in the LPBF of Fe and Zn alloys for biodegradable medical implants, particularly in areas of processing, microstructure, mechanical properties, corrosion, and biocompatibility. For Fe, optimizing the relationship between processing parameters (laser power, scan speed, and energy density) and densification is critical, as current studies report conflicting findings on the optimal conditions for achieving defect-free parts. Additionally, research is needed to understand the microstructural variability in Fe and FeMn alloys, especially regarding the influence of Mn on phase stability and grain formation. For Zn alloys, a more comprehensive mapping of energy density ranges and standardized processing atmospheres is necessary to address inconsistencies in part quality. The balance between grain size, densification, and hardness also requires further exploration, especially in ZnMg alloys, where the effects of alloying elements on mechanical properties are not fully understood. In terms of mechanical properties, more studies are needed on the impact of scaffold design and porosity on the strength and degradation rates of both Fe and Zn alloys. Corrosion behavior also presents a complex challenge, with discrepancies in how alloying elements and scaffold designs influence degradation rates, requiring standardized testing protocols and long-term studies. Finally, for biocompatibility, further research on standardized testing methods and dynamic in vivo conditions is needed to resolve conflicting results related to cytotoxicity, cell viability, and osseointegration, as well as to optimize alloy compositions for improved clinical performance.

## Figures and Tables

**Table 1:** Characteristics of bioresorbable metals Fe, Mg, and Zn.

	Amount in Human Organism (g)	Blood Serum Level	Daily Allowance	Young’s Modulus (GPa)	In Vitro Corrosion Rate (mm/Year)
Fe	5	5–17.6 g/L	10–20 mg	200	0.012
Mg	25	1.6–2.5 mg/dL	0.7 g	41–45	0.10 ± 0.07
Zn	2	60–120 µg/dL	12–15 mg	96	0.08
